# Selection Signatures in the First Exon of Paralogous Receptor Kinase Genes from the *Sym2* Region of the *Pisum sativum* L. Genome

**DOI:** 10.3389/fpls.2017.01957

**Published:** 2017-11-14

**Authors:** Anton S. Sulima, Vladimir A. Zhukov, Alexey A. Afonin, Aleksandr I. Zhernakov, Igor A. Tikhonovich, Ludmila A. Lutova

**Affiliations:** ^1^All-Russia Research Institute for Agricultural Microbiology, Saint-Petersburg, Russia; ^2^Department of Genetics and Biotechnology, Faculty of Biology, Saint-Petersburg State University, Saint-Petersburg, Russia

**Keywords:** pea (*Pisum sativum* L.), molecular evolution, legume–rhizobial symbiosis, LysM-containing receptor-like kinases, *Sym2*, Nod factor perception, *Medicago truncatula*

## Abstract

During the initial step of the symbiosis between legumes (Fabaceae) and nitrogen-fixing bacteria (rhizobia), the bacterial signal molecule known as the Nod factor (nodulation factor) is recognized by plant LysM motif-containing receptor-like kinases (LysM-RLKs). The fifth chromosome of barrel medic (*Medicago truncatula* Gaertn.) contains a cluster of paralogous LysM-RLK genes, one of which is known to participate in symbiosis. In the syntenic region of the pea (*Pisum sativum* L.) genome, three genes have been identified: *PsK1* and *PsSym37*, two symbiosis-related LysM-RLK genes with known sequences, and the unsequenced *PsSym2* gene which presumably encodes a LysM-RLK and is associated with increased selectivity to certain Nod factors. In this work, we identified a new gene encoding a LysM-RLK, designated as *PsLykX*, within the *Sym2* genomic region. We sequenced the first exons (corresponding to the protein receptor domain) of *PsSym37, PsK1*, and *PsLykX* from a large set of pea genotypes of diverse origin. The nucleotide diversity of these fragments was estimated and groups of haplotypes for each gene were revealed. Footprints of selection pressure were detected *via* comparative analyses of SNP distribution across the first exons of these genes and their homologs *MtLYK2, MtLYK3*, and *MtLYK4* from *M. truncatula* retrieved from the Medicago Hapmap project. Despite the remarkable similarity among all the studied genes, they exhibited contrasting selection signatures, possibly pointing to diversification of their functions. Signatures of balancing selection were found in LysM1-encoding parts of *PsSym37* and *PsK1*, suggesting that the diversity of these parts may be important for pea LysM-RLKs. The first exons of *PsSym37* and *PsK1* displayed signatures of purifying selection, as well as *MtLYK2* of *M. truncatula*. Evidence of positive selection affecting primarily LysM domains was found in all three investigated *M. truncatula* genes, as well as in the pea gene *PsLykX*. The data suggested that *PsLykX* is a promising candidate for *PsSym2*, which has remained elusive for more than 30 years.

## Introduction

One of the hallmark features of legumes (Fabaceae) is their ability to form beneficial symbioses with nitrogen-fixing soil bacteria collectively known as rhizobia. The establishment of such symbioses is a very complex process that involves many genes, as it requires proper recognition of the symbiotic partners, with subsequent development of novel symbiotic organs and partial integration of metabolic pathways in organisms belonging to different domains (Oldroyd et al., 2011; Suzaki et al., 2015; Zipfel and Oldroyd, 2017). The main function of these symbioses is the fixation of atmospheric nitrogen, making it a significant evolutionary advantage for legumes (Wheatley and Sprent, 2010; Werner et al., 2015).

The establishment of the legume–rhizobial symbiosis begins with mutual recognition of the partners. During this initial step, a bacterial lipo-chitooligosaccharide signaling molecule known as the Nod factor (nodulation factor; Denarie et al., 1996) is recognized by plant receptors in the LysM-RLK protein family (Limpens et al., 2003; Madsen et al., 2003; Broghammer et al., 2012). LysM-RLKs are receptor-like kinases (RLKs) with three lysin motifs (LysMs) in the ligand-binding extracellular region (Nakagawa et al., 2011; Mesnage et al., 2014). Although LysMs are widespread among prokaryotes and eukaryotes (with the exception of the Archaea), they are only connected to a kinase domain in plants (Ponting et al., 1999; Buist et al., 2008). Various LysM-RLKs are required to recognize molecular signals from rhizobia, arbuscular-mycorrhizal fungi (Buendía-Clavería et al., 2003; Oldroyd, 2013; Gobbato, 2015; Kawaharada et al., 2015; Buendia et al., 2016; Rasmussen et al., 2016), and some pathogenic microorganisms (Zhang et al., 2015). Thus, they occur also in non-legumes such as, *Arabidopsis thaliana*, which is unable to form nitrogen-fixing nodules or mycorrhizal associations (Veiga et al., 2013). Nonetheless, LysM-RLKs play a crucial role in regulating the legume–rhizobial symbiosis, providing plant selectivity toward microsymbiotic partners. Consequently, LysM-RLKs contribute to the selection of the most effective combinations of micro- and macrosymbionts (Provorov and Vorobyov, 2013).

Many genes encoding LysM-RLKs have been described in model legumes, such as, barrel medic (*Medicago truncatula* Gaertn; Arrighi et al., 2006), and *Lotus japonicus* (Regel) K. Larsen (Lohmann et al., 2010). Some are known to participate in symbiosis, while others have non-symbiotic functions. Interestingly, each legume species seems to have its own nuances in the organization and function of symbiotic LysM-RLKs; for example, mutations in the orthologous LysM-RLK genes *NFR1* (*NOD FACTOR RECEPTOR 1*) from *L. japonicus* and *MtLYK3* (*LysM DOMAIN-CONTAINING RECEPTOR-LIKE KINASE 3*) from *M. truncatula* manifested differently, blocking the penetration of rhizobia into plant cells at distinct stages of symbiotic development (Smit et al., 2007).

Garden pea (*Pisum sativum* L.) is a valuable pulse crop (http://www.fao.org/faostat). Studies on its symbiosis with rhizobia are very important (Borisov et al., 2004, 2007; Smýkal et al., 2012; Zhukov et al., 2016), since effective symbioses can increase the yield and quality of pea crops. Because of the long history of pea domestication and its wide distribution (Jing et al., 2010; Smýkal et al., 2012), many research centers around the world have large collections of pea genetic resources, making it possible to study the natural variability of particular pea genes and corresponding traits using sample sets close to genetic saturation (Jing et al., 2010). The N. I. Vavilov Institute of Plant Genetic Resources (VIR) in St. Petersburg, Russia, maintains a uniquely valuable collection of crop germplasm that includes pea and other legumes (Plekhanova et al., 2017). Studies on large ecotype collections and the use of association genetics may help uncover valuable alleles related to responses to environmental stresses (Gentzbittel et al., 2015). Recent genome-wide association (GWA) studies of the chickpea (*Cicer arietinum* L.) germplasm collection of the VIR revealed potential candidate genes likely to affect traits of agricultural importance (Plekhanova et al., 2017). Unfortunately, many molecular genetic methods are unsuitable for analyses of the pea genome because it is very large, congested with repetitive elements (Macas et al., 2007), without even a draft assembly. However, the high degree of synteny between the genomes of pea and *M. truncatula*, a well-studied model legume, makes it possible to investigate pea genes using the *M. truncatula* genome as a reference (Young and Udvardi, 2009).

The first discovered symbiosis-related pea gene was *PsSym2*, which determines increased selectivity toward rhizobia in the pea cv. Afghanistan (Govorov, 1928; Razumovskaya, 1937; Lie, 1984). Although the phenotype conferred by the allelic state of *PsSym2* is clear, the molecular basis of its function is not. It is thought to encode a LysM-RLK, because the selectivity inherent to cv. Afghanistan is associated with the ability to distinguish among the structural features of Nod factors produced by different (effective, ineffective, or even pathogen-like) rhizobial strains. Previously, *PsSym2* was localized in the linkage group I (LG I) of the pea genome (Kozik et al., 1996). Later, two paralogous genes encoding LysM-RLKs (*PsSym37* and *PsK1*) were found to co-localize there (Zhukov et al., 2008). However, neither of them was shown to play the same role as *PsSym2*, although both were shown to participate in bacterial signal recognition (Zhukov et al., 2008). In *M. truncatula*, the genomic region corresponding to the pea *PsSym2* location (*LYK* region) contains at least seven highly similar RLK genes; *MtLYK1*–*MtLYK7* (Limpens et al., 2003). One of these genes, *MtLYK3*, was identified as a symbiotic gene that controls bacterial penetration into the infection thread (Limpens et al., 2003; Smit et al., 2007). Considering these facts, one could expect to find more genes encoding RLKs in pea LG I, including genes related to symbiosis.

The early domestication and subsequent global dissemination of pea has led to significant and multifaceted selection pressure, resulting from both natural and breeding selection (Smýkal et al., 2012). Several loci associated with agronomically important traits have been found to play crucial roles in the domestication process (Weeden, 2007). However, the loci responsible for symbiosis, such as, bacterial recognition genes, are unlikely to be directly affected by breeding, although their influence on plant productivity should result in some selective pressure. Thus, the aim of this work was to estimate the genetic diversity of the genes encoding LysM-RLKs in the *Sym2* region of the pea genome and corresponding genes from the *LYK* region in the *M. truncatula* genome, and to evaluate and compare the selection pressures affecting different parts of the sequences. To this end, we (i) examined the region containing *PsSym37* and *PsK1* in pea LG I to identify new LysM-RLK genes; (ii) studied the polymorphism of the first exon sequences (corresponding to the receptor domain of the encoded protein) of three LysM-RLK genes (two known genes and one newly identified gene) in a large set of pea genotypes; and (iii) compared the selection signatures in pea genes with those in their *M. truncatula* homologs. As a result, we found a new gene, which we named *PsLykX* (for *P. sativum LysM kinase eXclusive*), and detected signatures of positive, balancing, and negative selection in the gene sequences. The polymorphism levels and patterns of selective signatures were unique to each of the three pea genes, but remarkably similar for the *Medicago* genes *MtLYK3* and *MtLYK4*. The results of the polymorphism analyses indicate that *PsLykX* is a promising candidate for *PsSym2*, the determinant of the unique symbiotic phenotype of cv. Afghanistan.

## Materials and methods

### Screening of the pea BAC library

To find novel LysM-RLK genes in the *Sym2* region, the pea Psa-B-Cam BAC library screening was performed at the INRA-CNRGV Plant Genomic Center (Toulouse, France). The screening was carried out via quantitative PCR, using BAC DNA pools amplified with Phi29 DNA polymerase and primer pairs for *PsSym37* and *PsK1* genes (Table S1). Information about the BAC library can be found online: (http://cnrgv.toulouse.inra.fr/).

**Figure 1:**
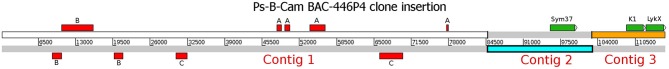
The assembled insertion in the Psa-B-Cam BAC-446P4 containing three LysM-RLK genes. Three contigs (Contig 1–Contig 3) were assembled. Genes *PsSym37, PsK1*, and *PsLykX* are represented by green rectangles. Transposable elements are represented by red rectangles. A, *OGRE* transposable element fragments; B, *Cyclops-2* transposable element fragments; C, *Ty3*-type retrotransposon gag-pol precursor pseudogene fragments.

The whole insertion in the identified BAC Psa-B-Cam 446P4 was sequenced using the 454 GS-Junior system with the Titanium kit in CNRGV, yielding 11697 reads with a mean length of 500 bp. Reads were then quality-trimmed using AdapterRemoval software (v. 2.1.7) (Lindgreen, 2012) and mapped onto the *Escherichia coli* DH5α strain genome using hisat2 mapper (Kim et al., 2015) to eliminate bacterial contamination. Then, 10,927 non-aligned reads were assembled using SPAdes (v 3.9.1) assembler (Bankevich et al., 2012), resulting in three contigs, which were uploaded to the NCBI database as a single entry under the accession number MF185734. Three genes (*PsSym37, PsK1*, and the novel gene *PsLykX*) were found via BLASTn searches (e-value cutoff 10^−6^) using the sequence of *PsK1* as the query (Figure 1). Additional BLAST searches against the NCBI non-redundant nucleotide database revealed fragmented transposable elements (Figure 1), but no additional genes.

The 3′-and 5′-ends of *PsLykX* cDNA were obtained by rapid amplification of cDNA ends (RACE) using total cDNA from inoculated roots of cv. Cameor. The Mint kit was used for cDNA synthesis and Mint kit primers were used for amplification of cDNA ends (Evrogen, Moscow, Russia). To obtain RNA for cDNA synthesis, five cv. Cameor plants were inoculated with *Rhizobium leguminosarum* bv. *viciae* strain RCAM 1026 and then grown in sterile sand for 10 days (Afonin et al., 2017). The specific primers used for RACE PCR are listed in Table S1.

### Plant material

The pea genotypes were previously selected by Borisov et al. (2002) from the collection of cultivated peas of the VIR (Saint Petersburg, Russia). The criterion for selection was diversity of places of origin. Preference was given to wild genotypes or primitive varieties, the so-called landraces. This set of 99 genotypes has already been tested for the efficiency of symbiosis with arbuscular-mycorrhizal (AM) fungi under the conditions of rhizobia inoculation (Jacobi et al., 2000; Borisov et al., 2002), and also for their responses to the toxic heavy metal cadmium under symbiotic conditions (Belimov et al., 2015). The originally obtained samples were propagated by selfing for several generations at the All-Russia Research Institute for Agricultural Microbiology (ARRIAM, Saint Petersburg, Russia). The genotypes and their places of origin (when known) are shown in Table S2.

### Plant DNA extraction

DNA was extracted from 3- to 4-day old seedlings of each pea genotype. The seedlings were cultivated in Petri dishes filled with sterile vermiculite at 28°C. Three to five seedlings per genotype were bulked and used for DNA extraction using the slightly modified CTAB protocol described elsewhere (Rogers and Bendich, 1985). Briefly, the steps were as follows: seedlings, 3–4 glass beads, and 700 μl 2 × CTAB buffer were placed into flat-bottomed microcentrifuge tubes and ground with the Fastprep-24 homogenizer (MP Biomedicals, Irvine, CA USA; 2 × 60 s at maximum frequency), then incubated in water bath for 2 h at 65°C. Then, 700 μl chloroform/isoamyl alcohol (24:1 v:v) was added to each tube, the mixture was vortexed, and then centrifuged for 10 min at 16,873 g. The upper fraction was transferred to a fresh microcentrifuge tube, and the previous step was repeated with 300–500 μl chloroform instead of chloroform/isoamyl alcohol. The upper fraction was again transferred to a fresh tube, and 1,000 μl 96% ethanol and 20 μl 5 M NaCl were added. The tubes were gently inverted two to three times to mix the contents, and then the mixture was centrifuged (10 min, 16,873 g). The supernatant was discarded, and 500 μl 70% ethanol was added to the pellet. The samples were vortexed, incubated at room temperature for 30 min, and then centrifuged (10 min, 16,873 g). The ethanol was discarded, and the tubes were incubated at 37°C for 20–30 min. Finally, the samples were dissolved in 20–30 μl TE buffer (pH 8.0). In total, DNA samples from 93 separate genotypes were used in further analyses.

### Sequencing of first exon of pea genes encoding LysM-RLKs

To amplify the first exons of *PsSym37, PsK1*, and *PsLykX*, a set of PCR primers was designed (Table S1). For different pea lines, different pairs of primers were used to ensure successful amplification. The quality of primers was verified by the OligoCalc on-line service: http://biotools.nubic.northwestern.edu/OligoCalc.html (Kibbe, 2007). The PCRs were performed in 96-well plates on an iCycler (Bio-Rad, Hercules, CA, USA) or Dyad (Bio-Rad, USA) instrument using the ScreenMix-HS kit (Evrogen, Moscow, Russia). The PCR cycling conditions were as follows: 95°C (5 min), 35 × [95°C (30 s), Tm (varying depending on primers) (30 s), 72°C (1 min)], 72°C (5 min). The PCR fragments were sequenced using the ABI Prism3500xL system (Applied Biosystems, Palo Alto, CA, USA) at the “Genomic Technologies, Proteomics, and Cell Biology” Core Center of the ARRIAM. The sequences of the first exons have been deposited in the NCBI under the accession numbers MF155289–MF155381 (*PsK1*), MF155382–MF155469 (*PsLykX*), and MF155470–MF155549 (*PsSym37*).

### Data collection from *M. truncatula* hapmap project

The Medicago Hapmap project, a long-term, community-accessible GWA mapping resource, is based on re-sequencing of 384 inbred lines spanning the range of *Medicago* diversity using Illumina next-generation sequencing technology (Stanton-Geddes et al., 2013). To study the polymorphisms of genes encoding LysM-RLKs, sequences of the first exons of *MtLYK2* (Medtr5g086310), *MtLYK3* (Medtr5g086130), and *MtLYK4* (Medtr5g086120) were retrieved from the Medicago Hapmap project website, assembly Mt4.0 (http://www.medicagohapmap.org/). Single nucleotide variants at particular sites were considered different from the reference *M. truncatula* genome if they were detected in more than 50% of corresponding Illumina reads. In total, 116 sequences for *MtLYK2*, 220 for *MtLYK3*, and 196 for *MtLYK4* were included in subsequent analyses.

### Pairwise comparison of LysM-RLKs

Pairwise comparison of all LysM receptor kinases was performed using the Needleman–Wunsch global alignment algorithm from the EMBOSS suite v.6.3.1 (Rice et al., 2000), using standard parameters. Comparisons of the three pea and three *M. truncatula* LysM-RLKs were performed separately for whole sequences, the kinase part, and the receptor part. The results from each comparison were combined using a custom python script. The results for full genes are shown in Table 1, and the results for the separate regions are shown in Tables S3, S4.

**Table 1:** Identity and similarity of *M. truncatula* MtLYK1-7, *P. sativum* PsSym37, PsK1 and PsLykX, *Lotus japonicus* LjNFR1 and *Arabidopsis thaliana* AtCERK1 whole putative proteins calculated by pairwise comparison.

	**AtCERK1**	**LjNFR1**	**MtLYK1**	**MtLYK2**	**MtLYK3**	**MtLYK4**	**MtLYK5**	**MtLYK6**	**MtLYK7**	**PsK1**	**PsLykX**	**PsSym37**
**IDENTITY**												
AtCERK1	100	55.1	47.6	54.2	54.8	46.7	48.8	53.7	55.7	54.6	48.6	54.8
LjNFR1	55.1	100	52	78.2	77.8	58.1	54.9	54.3	57.4	78.8	60.5	78.2
MtLYK1	47.6	52	100	51.9	50.6	64.5	72.1	57.7	56.5	51.7	61.6	51.6
MtLYK2	54.2	78.2	51.9	100	81	59.9	51.9	54.2	56.2	80.9	64.4	81.2
MtLYK3	54.8	77.8	50.6	81	100	66	50.5	54	57.2	84.4	63.5	83.7
MtLYK4	46.7	58.1	64.5	59.9	66	100	71.5	50.5	52.6	61.2	70	61.1
MtLYK5	48.8	54.5	72.1	51.9	50.5	71.5	100	55.3	58	53.3	64.5	53.2
MtLYK6	53.7	54.3	57.7	54.2	54	50.5	55.3	100	63.8	54.2	51.5	53.5
MtLYK7	55.7	57.4	56.5	56.2	57.2	52.6	58	63.8	100	57.6	52.7	57.7
PsK1	54.6	78.8	51.7	80.9	84.4	61.2	53.3	54.2	57.6	100	65	86.3
PsLykX	48.6	60.5	61.6	64.4	63.5	70	64.5	51.5	52.7	65	100	64.9
PsSym37	54.8	78.2	51.6	81.2	83.7	61.1	53.2	53.5	57.7	86.3	64.9	100
**SIMILARITY**												
AtCERK1	100	71.2	63	69	70.3	61.8	64.8	68.9	71.5	69.1	63.1	68.1
LjNFR1	71.2	100	65	86.8	86.3	71.1	66.8	68.5	71.9	87.1	72.2	86.6
MtLYK1	63	65	100	65.3	63.4	73.7	81.6	71	69.8	64.6	71.6	63.8
MtLYK2	69	86.8	65.3	100	89.5	73.6	67.1	69.7	71.7	90.2	76.2	88.7
MtLYK3	70.3	86.3	63.4	89.5	100	75.6	64.5	68.1	71.2	91.3	75.3	89.8
MtLYK4	61.8	71.1	73.7	73.6	75.6	100	80.9	65.6	65.9	73.1	79.9	74.1
MtLYK5	64.8	66.7	81.6	67.1	64.5	80.9	100	70.3	71.2	66.8	74.4	67.1
MtLYK6	68.9	68.5	71	69.7	68.1	65.6	70.3	100	75.6	69.1	65.9	66.4
MtLYK7	71.5	71.9	69.8	71.7	71.2	65.9	71.2	75.6	100	72.2	67.3	71.3
PsK1	69.1	87.1	64.6	90.2	91.3	73.1	66.8	69.1	72.2	100	76.3	92.4
PsLykX	63.1	72.2	71.6	76.2	75.3	79.9	74.4	65.9	67.3	76.3	100	76.2
PsSym37	68.1	86.6	63.8	88.7	89.8	74.1	67.1	66.4	71.3	92.4	76.2	100

*The “heat map” coloring indicates the degree of identity and similarity*.

Pairwise comparison of the known pea *Sym2* region and the *M. truncatula LYK* region was performed using BLAST and visualized using ACT software (release 13.0.0; Carver et al., 2005). The sequence of the *Sym2* region was obtained from the BAC Psa-B-Cam 446P4 clone (see Results). The *M. truncatula LYK* region sequence (g5:37100000–g5:537450000) was retrieved from the Phytozome website (www.phytozome.net/). The minimal identity cut-off was set to 70%. The exon/intron structure of genes in these regions was modeled by the Exonerate package (http://www.ebi.ac.uk/about/vertebrate-genomics/software/exonerate) with the -est2genome option enabled to search for gene mRNAs in the respective genomes.

### Polymorphism analysis and detection of selection pressures

Neighbor-joining (NJ) trees were constructed used MEGA v.6.60 software (Tamura et al., 2013) with the assumption that substitutions followed the Jukes–Cantor model and had uniform rates among sites. Bootstrap tests of NJ trees were performed with 500 bootstrap replications. The branches were cut off at 70% bootstrap support. Trees were visualized using FigTree (http://tree.bio.ed.ac.uk/software/figtree/).

We used DnaSP v5.10.01 (Librado and Rozas, 2009) to assess expected haplotype heterozygosity (HHe), and nucleotide diversity (π), including that for synonymous (π_s_) and non-synonymous (π_a_) sites, and to conduct the following neutrality tests: Tajima's D test (Tajima, 1989), Fu's Fs test (Fu, 1997), Fu and Li's D and H tests, Fay and Wu's H test (Fay and Wu, 2000), and McDonald-Kreitman MK test (McDonald and Kreitman, 1991). When needed, an outgroup sequence was chosen as follows: *PsSym37* (cv. Cameor) for barrel medic genes and *MtLYK3* (cv. Jemalong) for pea genes. DnaSP v5.10.01 was also used for sliding window analyses (100-bp window size, 25-bp step size), and to determine the significance of departure from the neutrality model by coalescent simulations with 1,000 replicates. Other neutrality tests, namely normalized Fay and Wu's H (nH) test (Zeng et al., 2006) and Ewens–Watterson (EW) test (Zeng et al., 2007), were carried out using DH software (http://zeng-lab.group.shef.ac.uk).

The codon-based Z-test considering the average rate of synonymous (*dS*) and nonsynonymous (*dN*) substitutions per site was performed in MEGA v.6.60 using the modified Nei–Gojobori method (Nei and Gojobori, 1986), with Jukes–Cantor correction (Jukes and Cantor, 1969) for multiple substitutions. Standard errors were estimated from 1,000 bootstrap replicates.

In all cases, separate estimates were made for the whole sequences of the coding part of the first exons and for sequences corresponding to LysM modules and intermediate parts. The borders of LysM modules were drawn as described previously (Zhukov et al., 2008).

### Inoculation experiment

An inoculation experiment was performed to determine the symbiotic phenotype of the pea line K-6883 (84). Seeds of K-6883 and cv. Cameor used as the inoculum control were planted in 2-L pots with sterile sand and inoculated with either strain RCAM1026 or A1 (Chetkova and Tikhonovich, 1986) of *R. leguminosarum* bv. *viciae*. Each pot contained five seeds, and the experiment was carried out with two technical replicates. After 28 days, the plants were removed from the pots and the phenotype of the root system was examined. The average number of nodules was calculated in SigmaPlot 12.0 (Systat Software, Inc., San Jose, CA, USA).

## Results

### Discovery of a new LysM-RLK gene in the *sym2* region using pea BAC library

To detect novel genes encoding LysM-RLKs in the *Sym2* region of pea LG I, the pea Psa-B-Cam BAC library was screened at the INRA-CNRGV Plant Genomic Center using quantitative PCR with primer pairs for *PsSym37* and *PsK1* (see section Materials and Methods). In the BAC clone Psa-B-Cam 446P4, we identified the two previously known LysM-RLK genes *PsSym37* and *PsK1* (used as probes), and a new LysM-RLK gene that we named *PsLykX* (for *LysM kinase eXclusive*; see Figure 1). No other BAC clones were detected during further screening with a part of *PsLykX* as the probe. This may indicate a lack of saturation of the Psa-B-Cam library in this genomic region.

**Figure 2:**
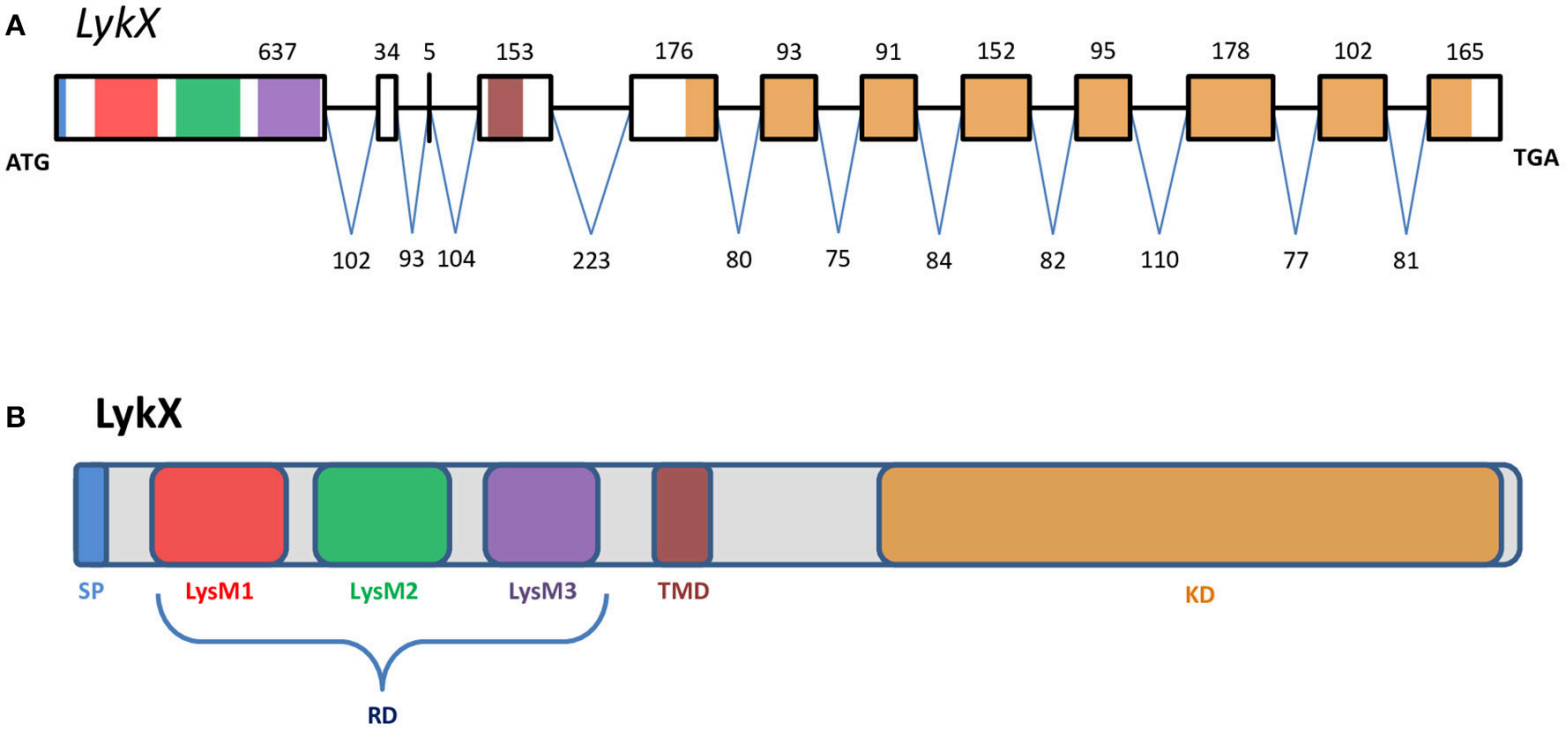
The exon-intron structure of the *PsLykX* gene **(A)** with domain structure of the corresponding LysM-RLK protein **(B)**. **(A)** Exons are designated by rectangles, introns—by horizontal lines. Numbers indicate the size of exons and introns. Colors match the colors of protein domains in **(B)** encoded by marked exons. **(B)** Colored blocks represent protein domains. SP, signal peptide; LysM1,2,3, LysM modules of receptor domain; RD, receptor domain; TMD, transmembrane domain; KD, kinase domain.

*PsLykX* was located close to *PsK1*; the stop codon of *PsK1* and the start codon of *PsLykX* were separated by 531 bp (Figure 1, also see MF185734 at GenBank). Despite this, *PsLykX* appeared to be a fully functional gene, because sequences perfectly matching its cDNA were found in pea transcriptome assemblies generated from nitrogen-fixing nodules (Alves-Carvalho et al., 2015; Sudheesh et al., 2015; Zhukov et al., 2015). The *PsLykX* complete open reading frame was successfully amplified from cDNA generated from RNA extracted from pea roots inoculated with nodule bacteria, and the sequences of *PsLykX* cDNA ends were verified using 3′- and 5′-RACE. Alignment of the genomic sequence and the cDNA of *PsLykX* revealed a gene structure similar to that of *PsSym37*, with 12 exons and 11 introns (Zhukov et al., 2008), which is typical of symbiotic LysM-RLK genes (Figure 2A). The sequence of *PsLykX* has been deposited in GenBank under the accession number MF135533.

**Figure 3:**
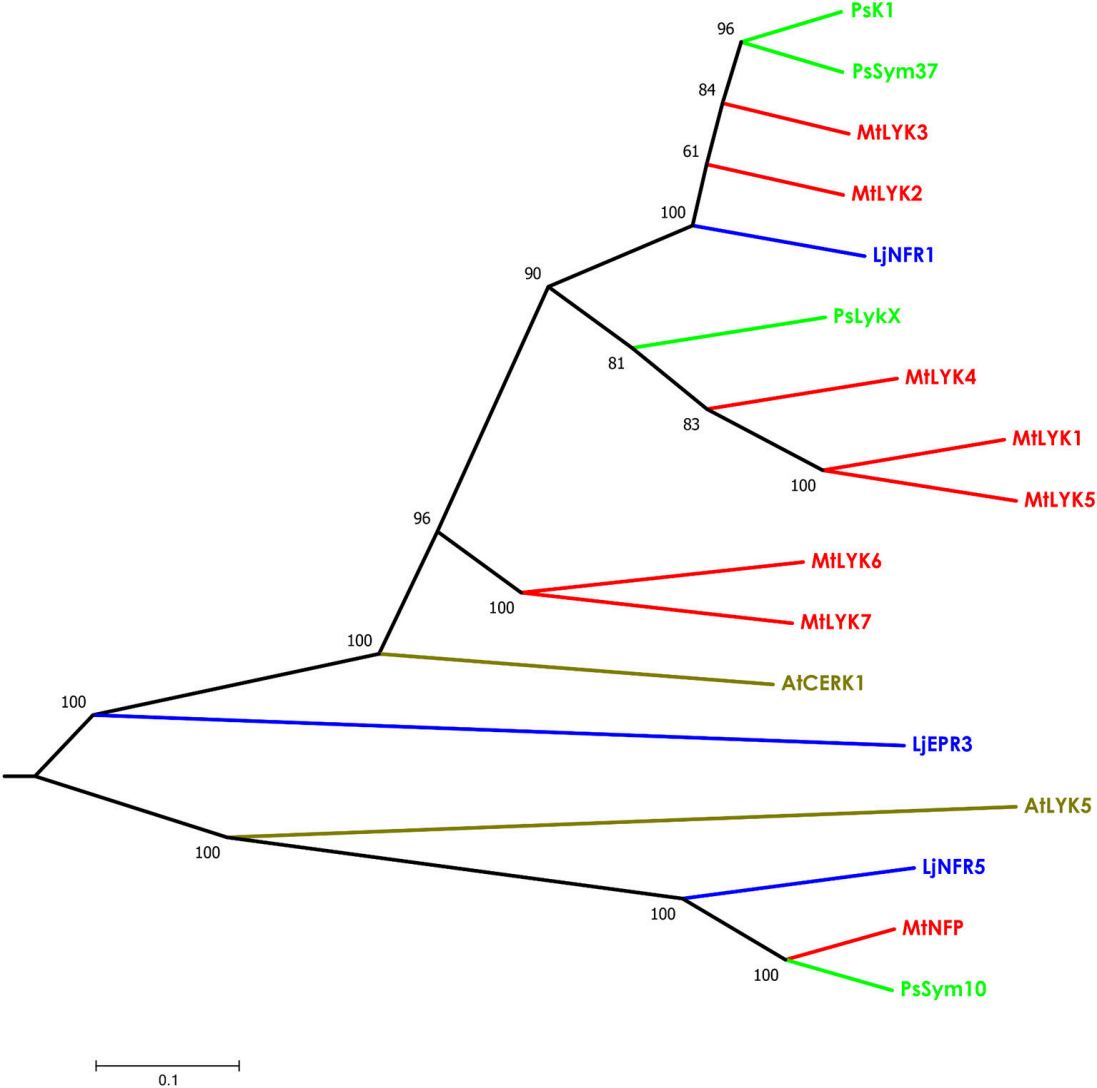
Phylogenetic tree showing the comparison of the whole amino acid sequences of LysM-RLKs from *Sym2* region of pea genome, LysM-RLKs from syntenic *LYK* region of *Medicago truncatula* genome, and homologous LysM-RLKs from *Lotus japonicus* and *Arabidopsis thaliana*. Branches are colored as follows: pea (Ps)—green, *M. truncatula* (Mt)—red, *Lotus japonicus* (Lj)—blue, *Arabidopsis thaliana* (At)—olive. Tree was built using the Neighbor-Joining method with the assumption that substitutions followed the Jukes–Cantor model and had uniform rates among sites. Bootstrap test of NJ tree was performed with 500 bootstrap replications.

The putative PsLykX protein contained three LysM modules encoded by the first exon and forming the receptor domain, along with transmembrane and kinase domains (Figure 2B). Multiple alignment between PsLykX and known LYKs from the syntenic *LYK* region of the *M. truncatula* genome and LysM-RLKs from other organisms revealed that PsLykX was closest to MtLYK4, MtLYK1 and MtLYK5 (Figure 3). In the neighbor-joining phylogenetic tree, PsLykX and MtLYK4 were grouped apart from PsSym37 and PsK1 which formed a distinctive clade with MtLYK2 and MtLYK3 of *M. truncatula* and symbiotic LysM-RLK NFR1 of *L. japonicus* (Figure 3).

### Polymorphism of first exon sequences of LysM-RLK genes in *sym2* region

To assess the level of polymorphism in parts of pea LysM-RLK genes encoding ligand binding structures, we sequenced the first exons (corresponding to the receptor domain) of the three identified LysM-RLK genes from the *Sym2* region in a subset of cultivated pea accessions (Table S2). To fully characterize the extent of polymorphism, previously obtained sequences of *PsSym37* and *PsK1* (Zhukov et al., 2008) available from online databases were included in the analysis. Overall, 101 sequences were analyzed for *PsK1*, 90 sequences for *PsLykX*, and 89 sequences for *PsSym37* (Table S2).

**Figure 4:**
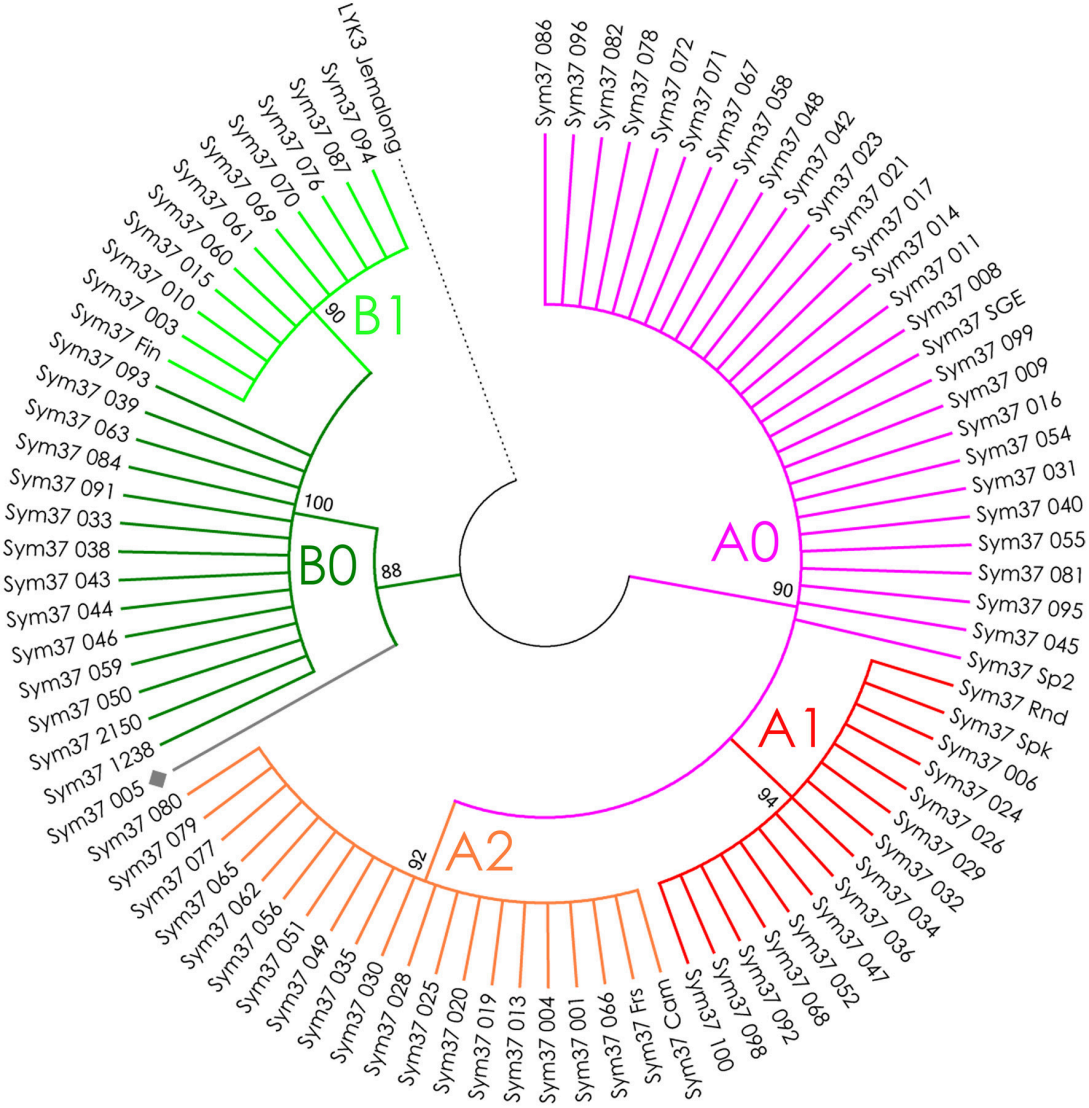
Phylogenetic tree representing the variability of *PsSym37* gene. Groups of haplotypes are labeled in accordance with the Table 2. Unique haplotypes are additionally marked with gray rhombuses. The *M. truncatula* cv. Jemalong gene *MtLYK3* was chosen as the outgroup.

**Figure 5:**
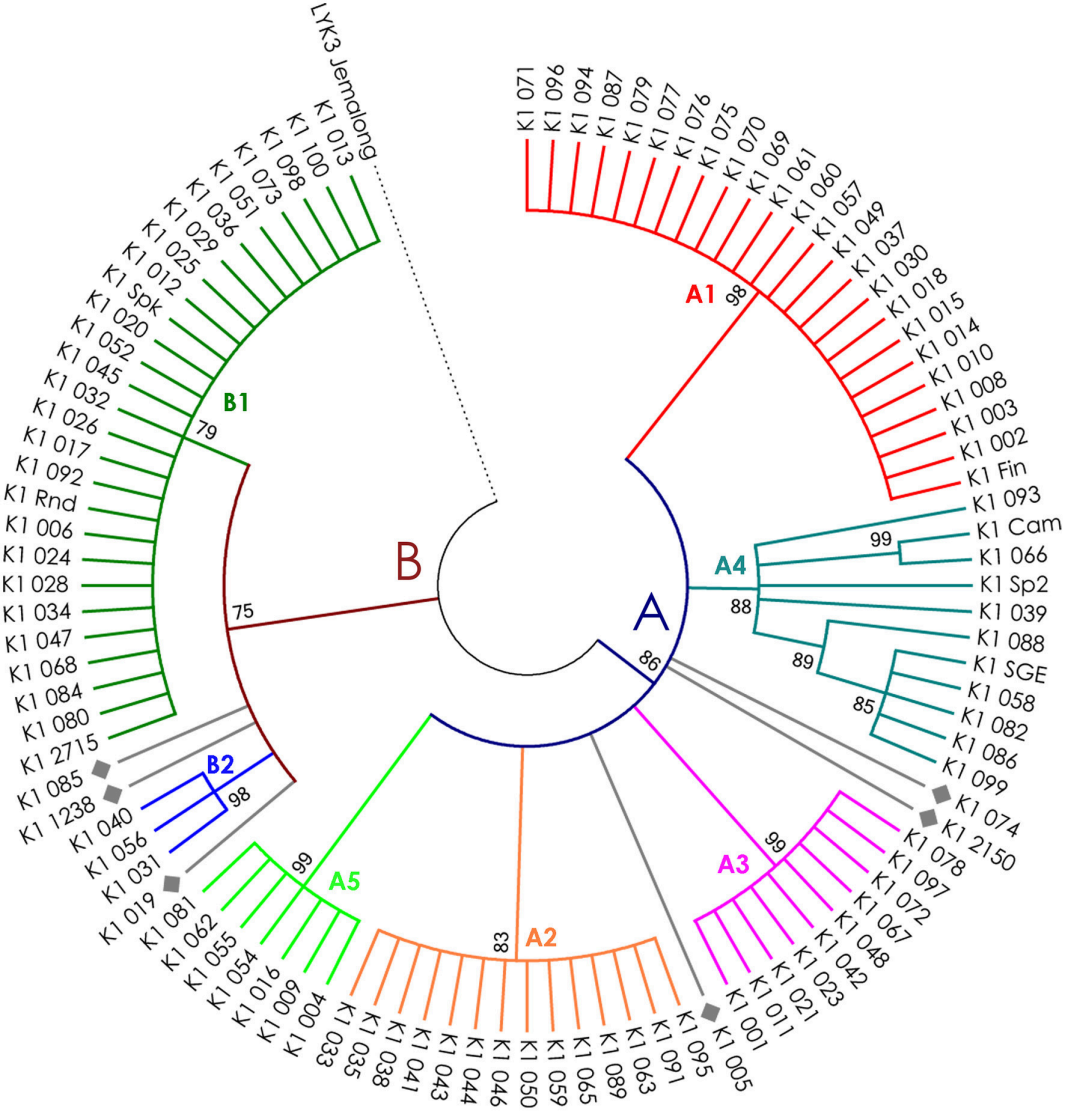
Phylogenetic tree representing the variability of *PsK1* gene. Groups of haplotypes are labeled in accordance with the Table 2. Unique haplotypes are additionally marked with gray rhombuses. The *M. truncatula* cv. Jemalong gene *MtLYK3* was chosen as the outgroup.

**Figure 6:**
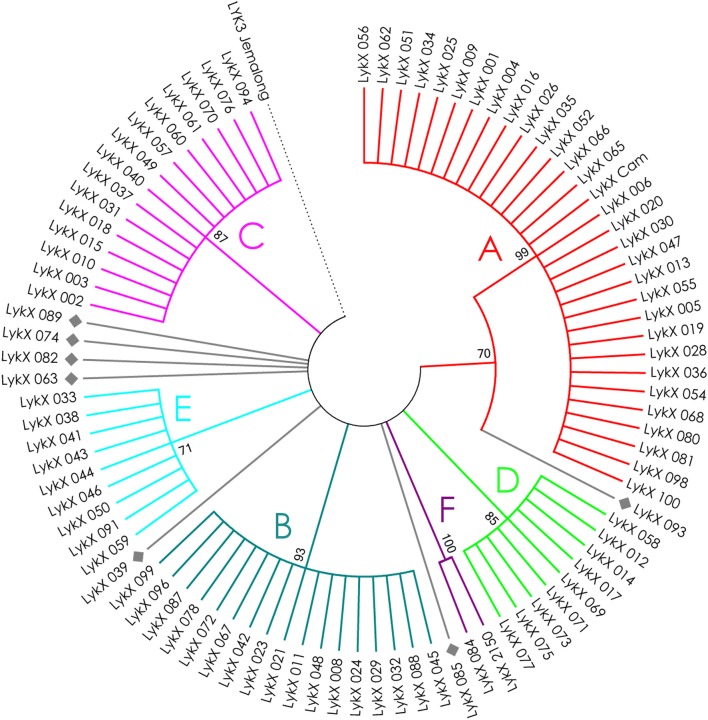
Phylogenetic tree representing the variability of *PsLykX* gene. Groups of haplotypes are labeled in accordance with the Table 2. Unique haplotypes are additionally marked with gray rhombuses. The *M. truncatula* cv. Jemalong gene *MtLYK3* was chosen as the outgroup.

The nucleotide sequences of the first exons were aligned using the codon-based ClustalW algorithm, and phylogenetic trees based on LysM-RLKs variability were constructed (Figures 4–**6** for *PsSym37, PsK1*, and *PsLykX*, respectively). The clades of the resulting trees were considered to represent distinct haplotypes. Comparison of amino acid sequences confirmed the haplotype distribution pattern (Table 2). *PsSym37* and *PsK1* sequences formed two main groups, each with several subgroups (Figures 4, 5), while *PsLykX* sequences appeared to be more diverse and formed six distinct groups (Figure 6).

**Table 2:** Haplotypes of pea LysM-RLKs.

***K1***																			
	**13**	**31**	**38**	**45**	**46**	**48**	**49**	**50**	**55**	**61**	**65**	**81**	**90**	**104**	**105**	**124**	**181**		
A1	F	L	V	P	T	K	N	Y	I	D	S	F	E	T	T	S	N		
A2	F	L	I	A	T	K	N	Y	I	D	S	F	D	T	T	T	N		
A3	F	L	V	P	T	K	N	Y	I	D	S	F	E	T	A	T	I		
A4	F	L	V/I	P	T	K	N/S	Y	I	N	S	F/S	E	I	T	S/T	N		
A5	F	L	V	P	T	K	N	Y	I	D	R	F	D	T	T	S	N		

B1	L	I	V	S	N	T	T	F	L	D	R	F	E	T	T	S	N		
B2	L	I	V	S	N	T	T	F	L	D	R	F	E	T	T	S	N		

K1 005	F	I	I	A	T	K	S	Y	I	D	S	F	E	T	T	T	I		
K1 019	F	I	V	S	N	T	T	F	I	D	R	F	E	T	T	T	K		
K1 074	F	L	V	P	T	K	N	Y	I	D	S	F	E	T	T	T	N		
K1 085	L	I	V	S	N	T	T	F	L	D	R	F	E	T	A	S	N		
K1 1238	L	I	V	S	N	T	T	F	L	D	R	F	E	T	T	T	N	158Q:R	172S:F
K1 2150	F	L	V	P	T	K	N	Y	I	D	S	S	E	T	T	T	N		
***Sym37***																			
	**43**	**45**	**56**	**60**	**83**	**131**	**141**	**181**	**184**	**205**									
A0	E	S	L	S	I	F	A	S	K	I									
A1	E	L	V	S	I	F	A	S	K	I									
A2	E	S	L	S	I	F	A	S	K	I									

B0	Q	S	V	F	V	Y	V/A	R	K	V									
B1	Q	S	V	F	V	Y	V	R	N	V									

Sym37 005	Q	S	V	S	I	F	A	R	K	V	26V:L								
***LykX***																			
	**9**	**13**	**16**	**18**	**23**	**42**	**44**	**45**	**75**	**76**	**82**	**86**	**111**	**134**	**142**	**184**			
A	L	L	V	F	K	L	Q	N	R	A	F	V	T	S	A	F			
B	F	V	V	F	K	K	Q	N	R	A	F	I	T	S	I	S			
C	L	L	F	S	K	K	Q	N	R	A	S	I	S	S	I	S			
D	L	V	F	F	K	K	Q	N	R	A	F	I	T	G	A	S			
E	L	L	F	S	K	K	Q	N	R	A	S	I	S	S	A	S			
F	L	L	V	F	K	K	R	Y	R	D	F	V	T	S	I	S			

LykX 039	L	L	V	F	K	K	Q	N	P	A	F	I	T	S	A	S	128V:F		
LykX 063	L	L	F	S	Q	K	Q	N	R	A	S	I	T	S	A	S			
LykX 074	L	V	F	S	K	K	Q	N	R	A	S	I	T	S	I	S	135H:D		
LykX 082	L	L	F	S	Q	K	Q	N	R	A	S	I	T	G	A	S	191I:M		
LykX 085	L	L	V	F	K	K	Q	N	P	A	F	I	T	S	I	S			
LykX 089	L	L	F	S	K	K	Q	N	R	A	S	I	S	S	I	S			
LykX 093	L	L	V	F	K	L	Q	S	R	A	F	I	T	S	A	S	5F:L	183A:T	

*Numbers indicate the amino acid position in sequence of corresponding protein. Letters indicate groups of haplotypes, as shown in Figures 4–6. Colors indicate functional parts of corresponding protein (blue—signal peptide, gray—interdomain region, red—LysM1, green—LysM2, violet—LysM3; also see Figure 2B)*.

The amino acid alignment analyses showed that *PsLykX* was the most variable among the three genes, with 16 amino acid polymorphisms in the putative protein sequence. According to these, the whole sample was divided into 13 groups, seven of which were represented by single unique genotypes with additional polymorphic sites. *PsK1* had 17 amino acid polymorphisms in the putative protein sequence, dividing sample into 12 groups (with six single-genotype groups). Interestingly, although group B of *PsK1* split into two subgroups, B1 and B2, according to nucleotide-based phylogeny, the haplotypes in these subgroups were identical at the amino acid level. *PsSym37* appeared to be more uniform, with only six groups based on 10 amino acid polymorphisms (with one group being represented by a sole genotype).

In all three genes, the most polymorphic region was the sequence corresponding to the first LysM domain (LysM1); it contained the majority of non-synonymous substitutions (Table 2). In *PsK1* and *PsLykX*, several non-synonymous variations were detected in the part corresponding to the signal peptide (SP) as well, although these changes were unlikely to affect protein localization. Further research is required to explore the possible functional significance of these changes.

We identified *PsLykX* as a new gene from the *Sym2* region encoding an LysM-RLK. This gene is a promising candidate for the *PsSym2* gene, which is responsible for increased selectivity toward rhizobia in several genotypes of pea originating from Afghanistan and neighboring areas (Lie, 1984; Kozik et al., 1995). Plants carrying the so-called “Afghan” allele of *PsSym2* (*Sym2*^*A*^) form nodules only with strains producing a double-acetylated Nod factor, while plants with the most common “European” allele (*Sym2*^*E*^) are able to perceive the mono-acetylated Nod factor as well (Davis et al., 1988). Bearing this in mind, we sequenced the first exon of *PsLykX* of cv. Afghanistan (NGB2150), and found that it contained a unique haplotype of *PsLykX*, which was shared with only one other line, K-6883 (84). We investigated the symbiotic phenotype of K-6883 in an experiment with two strains of *R. leguminosarum* bv. *viciae*: RCAM1026 (Afonin et al., 2017), which produces a mono-acetylated Nod factor, and A1, which produces a double-acetylated Nod factor (Chetkova and Tikhonovich, 1986; Ovtsyna et al., 1999). K-6883 failed to form nodules with RCAM1026 but formed 105.0 ± 22.3 nodules with A1, while cv. Cameor (control belonging to another *PsLykX* haplotype) formed 151.6 ± 14.1 nodules with RCAM1026 and 105.2 ± 11.7 nodules with A1. Thus, K-6883 showed “Afghan” selectivity toward rhizobia, providing further evidence that *PsLykX* may actually be *PsSym2*.

### Identification of selection signatures

#### Investigation into the correspondence between pea and *M. truncatula* LysM-RLK genes

Polymorphism analyses can provide insights into the evolutionary pathways leading to the contemporary state of genes in related species. The fact that pea and barrel medic LysM-RLK genes are paralogs located close to each other made it difficult to estimate selection signatures in their sequences. The *Sym2* region of pea LG I is syntenic to the *M. truncatula LYK* region on chromosome 5 (Gualtieri et al., 2002), where seven LysM-RLK genes are located (Limpens et al., 2003), while only three genes are known to be present in the *Sym2* region of the pea genome. Thus, to compare the diversity of the pea LysM-RLK genes with that of the *M. truncatula* ones, we first determined the relationship between *PsSym37, PsK1*, and *PsLykX* and the *M. truncatula LYK* genes. To this end, we compared the whole sequences of LysM-RLK putative proteins (three from pea, seven from *Medicago*), as well as their receptor and kinase domains separately (Table 1, Tables S3, S4). We included *LjNFR1* as an ortholog of *PsSym37* and *MtLYK3* (Zhukov et al., 2008), and *AtCERK1* as non-symbiotic LysM-RLK of a distant species in these comparisons. The identity (strict matching of amino acids in corresponding positions) and similarity (functional analogy of different amino acids in corresponding positions) percentages were taken into account.

In addition, the whole BAC clone Psa-B-Cam 446P4 insertion was compared to the LYK region of the *M. truncatula* genome ver. 4.0 (chr5:37,100,000–37,450,000) using BLAST. The beginning of *PsSym37* showed very strong similarity to *MtLYK3*, even in the upstream region containing the putative promoter (Figure S1A). Exon 2, exon 3, and exons 6–8 of *PsSym37* were similar to corresponding exons in both *MtLYK2* and *MtLYK3*, while exon 5 and exon 11 were similar only to exon 5 and exon 11 of MtLYK3, and exon 4 was similar only to exon 4 of *MtLYK2*. This, together with phenotypic data obtained for mutants (Smit et al., 2007), corroborates the idea that *PsSym37* is the most likely ortholog of *MtLYK3*. For the remaining two pea genes, the similarity patterns were more complicated. In *PsK1*, exons 6–8 and exon 10 were similar to the corresponding exons in *MtLYK3* and *MtLYK2*, while exon 9 was similar only to exon 9 of *MtLYK2* (Figure S1B). Exons 6–9, 10, and 11 of *PsLykX* were similar to the uncharacterized transcribed sequence (Mtr.51442.1.S1_at at MtGEA v. 3.0; https://mtgea.noble.org/v3/ He et al., 2009) in the *LYK* region of *M. truncatula* (see Figure S1C), annotated in Mt4.0 as *Medtr5g086080*. However, exons 6–8, exon 10, and exon 11 were also similar to respective exons in *MtLYK4*. It is important to note that the first exon showed significant uniformity (at least 80%) both among the genes in one species (pea or *M. truncatula*) and between genes in different species (Table S3).

Our observations confirmed the previous finding that *PsSym37* is the most likely ortholog of *MtLYK3* (Zhukov et al., 2008). *PsLykX* was more similar to *MtLYK4* than to other *LYK* genes, whilst *PsK1* showed high similarity to both *MtLYK3* and *MtLYK2* (and also to *PsSym37*) at the putative protein level (Table 1). Taking into consideration the mosaic nature of genes in both the *LYK* and *Sym2* regions, the complex evolutionary history of these genes, and the possibility of concerted evolution of paralogous genes, we are cautious to postulate orthology between pea and *M. truncatula* LysM-RLK genes, aside from *PsSym37* and *MtLYK3*, which have conserved their basic function of Nod factor recognition and downstream signal transduction. Based on this, and since the first exons of all analyzed genes were >80% identical, we chose outgroups for polymorphism analyses as follows: *PsSym37* for all barrel medic genes, and *MtLYK3* for all pea genes.

The receptor parts of LysM-RLK contain three functionally important modules (LysM domains). Therefore, we assessed overall polymorphism in the first exon and polymorphism of separate parts of the first exon encoding LysM domains, as well as intermediate parts.

#### Codon-based tests for selection signatures

The ratio between synonymous and non-synonymous substitutions is often used to detect negative, or purifying, selection (i.e., selection against non-synonymous changes). The codon-based Z-test relies on a statistically significant difference between the rate of synonymous substitutions per synonymous site (*dS*) and the rate of non-synonymous substitutions per non-synonymous site (*dN*). We applied this test to the full sequences and to separate LysM module-encoding parts of the first exon of three pea and three *M. truncatula* genes. The results are shown in Table 3.

**Table 3:** Results of codon-based Z-test for examined regions of pea and *M. truncatula* LysM-RLK genes.

**Gene**	**Part**	**Z**
*PsK1*	Overall	−3.323^**^ **(Purifying)**
	Upstream LysM1	**–**1.315
	LysM1	−1.392
	Int. LysM1-2	1.131
	LysM2	−2.028^*^ **(Purifying)**
	Int. LysM2-3	2.372^*^ **(Positive)**
	LysM3	**–**1.715
*PsSym37*	Overall	−4.117^***^ **(Purifying)**
	Upstream LysM1	−2.163^*^ **(Purifying)**
	LysM1	−0.691
	Int. LysM1-2	**–**1.109
	LysM2	−2.263^*^ **(Purifying)**
	Int. LysM2-3	**–**1.903
	LysM3	**–**1.759
*PsLykX*	Overall	**–**1.827
	Upstream LysM1	**–**0.141
	LysM1	0.178
	Int. LysM1-2	**–**0.973
	LysM2	**–**1.083
	Int. LysM2-3	–
	LysM3	**–**1.619
*MtLYK2*	Overall	−3.306^**^ **(Purifying)**
	Upstream LysM1	**–**1.183
	LysM1	**–**1.487
	Int. LysM1-2	**–**1.098
	LysM2	−2.025^*^ **(Purifying)**
	Int. LysM2-3	**–**0.39
	LysM3	**–**1.934
*MtLYK3*	Overall	**–**0.389
	Upstream LysM1	−0.912
	LysM1	1.632
	Int. LysM1-2	–
	LysM2	**–**0.895
	Int. LysM2-3	**–**0.049
	LysM3	**–**0.239
*MtLYK4*	Overall	0.617
	Upstream LysM1	1.312
	LysM1	1.904
	Int. LysM1-2	**–**
	LysM2	**–**0.33
	Int. LysM2-3	1.286
	LysM3	**–**0.587

Values of Z-statistic showing the statistically significant difference from the neutral model (meaning that dN ≠ dS) are given in bold, with asterisks marking the significance level according to P-value:

* P < 0.05;

** P < 0.01;

*** *P < 0.001. The direction of the selection (purifying or positive) is given in brackets*.

The overall sequences of the first exons of *PsK1* and *PsSym37* showed a clear departure from neutrality in favor of purifying selection, while the first exon of *PsLykX* showed a tendency toward purifying selection (Table 3). Analysis of separate modules indicated that in both *PsK1* and *PsSym37*, the part encoding the LysM2 module (and, for *PsSym37*, also the part preceding LysM1 module) has undergone strong purifying selection. In *PsK1*, the intermediate part between LysM2 and LysM2-encoding regions showed signs of positive selection. There was also a tendency toward purifying selection in LysM3 and between LysM2 and LysM3 of *PsSym37*, which may indicate the importance of this gene. Indeed, mutations in *PsSym37* were shown to markedly decrease the number of nodules in symbiotic conditions (Zhukov et al., 2008). In addition, the site between LysM2 and LysM3 of *PsK1* appeared to be under positive selection, suggesting that the structure of LysM modules is more important than that of intermediate regions in case of this particular LysM-RLK.

In *Medicago, MtLYK2* has undergone purifying selection, which has strongly affected LysM2; this was also the case for *PsK1* and *PsSym37*. It is important to note that the function of *MtLYK2* is unknown; however, its similarity to *MtLYK3* and the results of our analyses suggest that *MtLYK2* may participate in the same symbiotic signal cascade as MtLYK3. However, *MtLYK3* clearly demonstrates neutral evolution, even though it is essential for Nod factor perception (Smit et al., 2007). A possible explanation is that *MtLYK3* has a low basic level of polymorphism (Table 4), which results in a statistically insignificant difference between *dN* and *dS*. *MtLYK4* has undergone neutral evolution, like *MtLYK3* and pea *PsLykX*. However, the neutral evolution signal may be a result of interference of two oppositely directed signals implying positive or purifying selection in different lineages in the dataset.

**Table 4:** Nucleotide diversity in examined regions of pea and *M. truncatula* LysM-RLK genes.

**Gene**	**Part**	**Length. bp**	**Segr. sites**	**HHe**	π^*^10^3^	$\pi_{\text{s}}^{*}$10^3^	$\pi_{\text{a}}^{*}$10^3^	**π_a_/π_s_**
*PsK1*	Overall	633	56	0.874	21.57	49.11	13.64	0.278
	Upstream LysM1	105	11	0.748	19.20	46.52	11.39	0.245
	LysM1	159	19	0.815	42.04	71.36	33.30	0.467
	Int. LysM1-2	42	2	0.346	8.33	0.00	10.40	–
	LysM2	141	11	0.830	22.71	68.46	9.16	0.134
	Int. LysM2-3	54	4	0.058	1.45	0.00	1.87	–
	LysM3	126	8	0.874	10.33	37.37	2.76	0.074
*PsSym37*	Overall	633	39	0.862	18.39	52.62	8.46	0.161
	Upstream LysM1	105	6	0.788	11.14	53.13	0.27	0.005
	LysM1	159	9	0.777	20.30	29.28	17.66	0.603
	Int. LysM1-2	42	2	0.320	7.81	37.72	0.00	0.000
	LysM2	141	10	0.675	19.39	59.87	6.72	0.112
	Int. LysM2-3	54	4	0.455	26.30	104.13	3.96	0.038
	LysM3	126	8	0.483	21.88	59.32	10.98	0.185
*PsLykX*	Overall	636	42	0.841	15.60	29.52	11.65	0.395
	Upstream LysM1	105	10	0.753	22.48	24.36	21.97	0.902
	LysM1	165	12	0.709	14.94	13.02	15.50	**1.190**
	Int. LysM1-2	42	1	0.313	7.44	37.51	0.00	0.000
	LysM2	141	10	0.802	20.70	42.42	14.06	0.331
	Int. LysM2-3	54	0	0.000	0.00	0.00	0.00	–
	LysM3	126	9	0.780	15.32	52.02	5.11	0.098
*MtLYK2*	Overall	639	65	0.915	12.74	30.38	7.69	0.253
	Upstream LysM1	105	9	0.439	14.25	28.52	10.46	0.367
	LysM1	165	16	0.729	14.16	27.32	10.49	0.384
	Int. LysM1-2	42	1	0.051	1.22	5.70	0.00	0.000
	LysM2	141	21	0.329	12.80	27.97	8.24	0.295
	Int. LysM2-3	54	3	0.163	4.24	5.99	3.77	0.629
	LysM3	126	15	0.816	17.50	58.75	5.69	0.097
*MtLYK3*	Overall	636	63	0.976	11.46	13.41	10.89	0.812
	Upstream LysM1	105	9	0.447	6.20	13.04	4.25	0.326
	LysM1	162	28	0.899	17.73	5.17	21.45	**4.149**
	Int. LysM1-2	42	0	0.000	0.00	0.00	0.00	–
	LysM2	141	9	0.744	22.13	40.14	16.84	0.420
	Int. LysM2-3	54	5	0.089	2.18	2.28	2.15	0.943
	LysM3	126	11	0.281	3.55	4.15	3.38	0.814
*MtLYK4*	Overall	639	47	0.971	12.21	12.26	12.19	0.994
	Upstream LysM1	105	5	0.391	5.71	2.57	6.61	**2.572**
	LysM1	162	13	0.562	10.91	1.58	13.78	**8.722**
	Int. LysM1-2	42	0	0.000	0.00	0.00	0.00	–
	LysM2	141	12	0.712	22.40	35.32	18.60	0.527
	Int. LysM2-3	54	3	0.564	12.23	0.00	15.57	–
	LysM3	126	13	0.697	10.30	18.61	8.01	0.430

*π_a_**/**π_s_ ratio higher than 1 is indicated in bold*.

We also used the McDonald–Kreitman test (MK-test) as a statistical test of synonymous and non-synonymous changes. This test compares genes in two related species. The MK test did not detect departure from a neutral model, with the exception of the LysM1 region for the gene pair *PsLykX* and *MtLYK4* (*P* = 0.0492).

#### SNP-based tests

We used several molecular evolution tests to analyze the distribution of SNP sites across the dataset, regardless of whether they represented synonymous or non-synonymous changes. These tests can identify either balancing selection (in favor of two or more alleles) or positive selection (in favor of one allele) and distinguish these types of selection from the neutral evolution model. First, nucleotide diversity (Pi, or π) was assessed in all sequences of the first exons and their separate parts, considering changes in synonymous and non-synonymous sites. In general, the rate of π was higher in pea genes than in *M. truncatula* ones (Table 4), even though the *M. truncatula* sequence dataset was larger than the pea dataset. In addition, haplotype diversity was higher for *M. truncatula* genes than for pea ones, possibly reflecting the prevalence of rare nucleotide variants (singletons) in the *Medicago* sequence dataset. The π_a_/π_s_ ratios for LysM1 in MtLYK3 and MtLYK4 were very high (4.1 and 8.7, respectively), indicating an excess of non-synonymous substitutions. This result indicated that natural selection has tended to support new variants of LysM1 in these genes.

**Figure 7:**
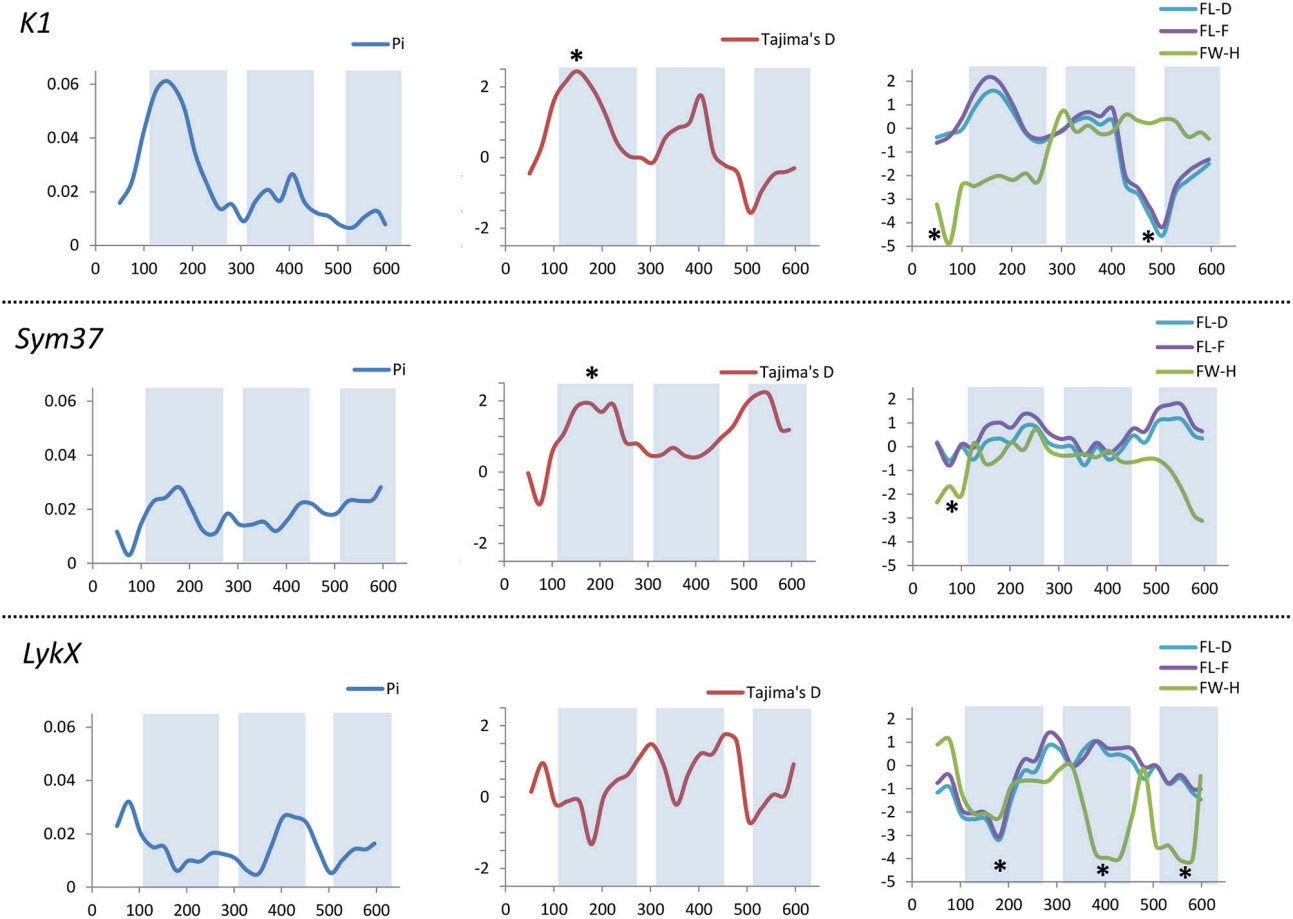
Sliding window analysis of nucleotide diversity and neutral evolution criteria in first exons of *PsSym37, PsK1* and *PsLykX* genes. LysM-encoding parts are highlighted. Statistically significant deviations from the neutral evolution model are marked with asterisks.

**Figure 8:**
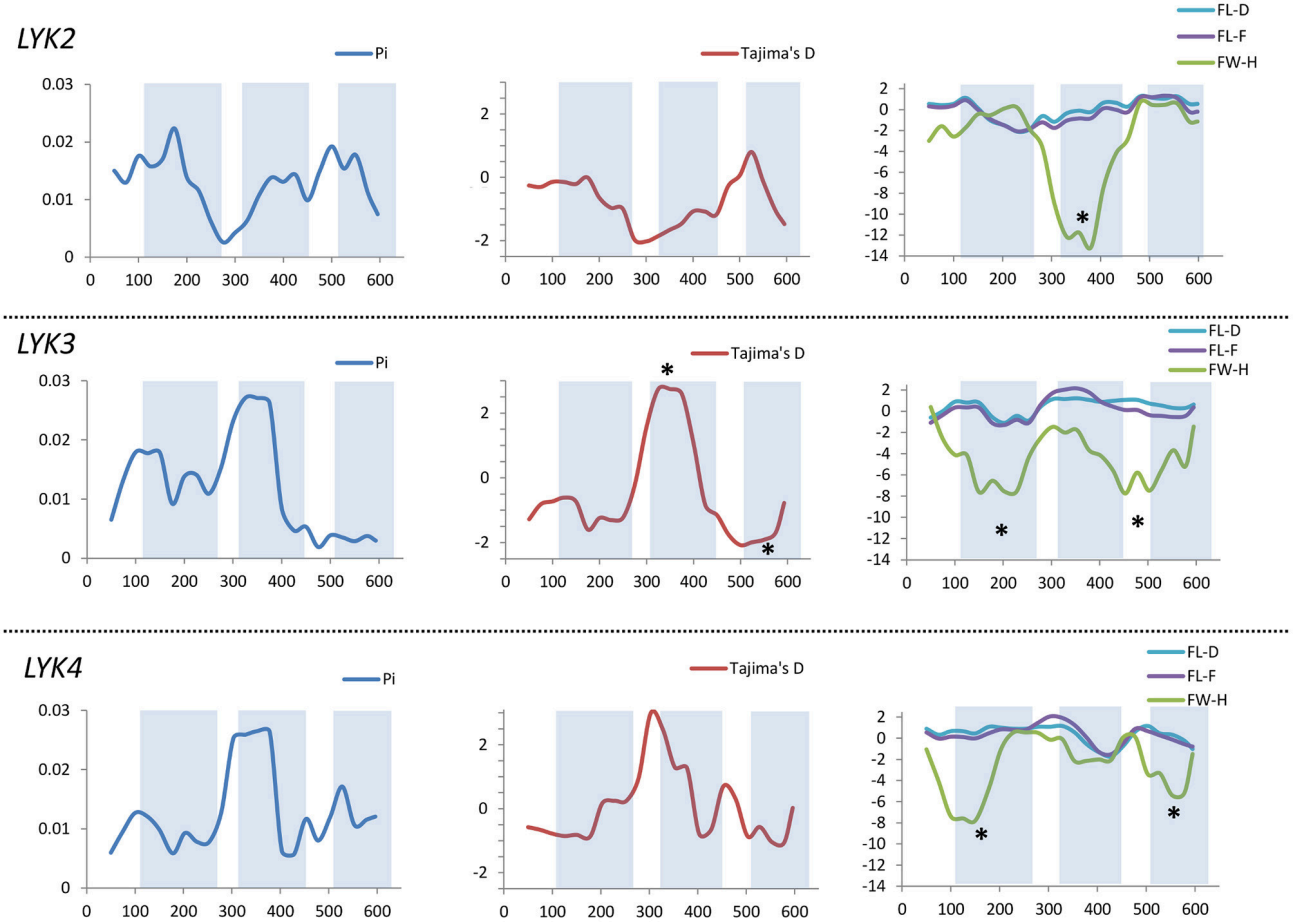
Sliding window analysis of nucleotide diversity and neutral evolution criteria in first exons of *MtLYK2, MtLYK3* and *MtLYK4* genes. LysM-encoding parts are highlighted. Statistically significant deviations from the neutral evolution model are marked with asterisks.

All the sequence sets were subjected to several neutrality tests (see section Materials and Methods, Table 5). In addition to analyses of the whole sequences and parts corresponding to LysM modules, a sliding window approach was used to visualize the obtained criteria values (Figures 7, 8).

**Table 5:** Results of the molecular evolution tests applied to the examined regions of pea and *M. truncatula* LysM-RLK genes.

**Gene**	**Part**	**Tajima's D**	**Fu's Fs**	**FL-D**	**FL-F**	**FW-H**	**nH**	**EW**
PsK1	Overall	0.649	1.664	−1.133	−0.577	−7.350	–1.904^*^	0.137
	Upstream LysM1	−0.123	−2.087	−0.208	−0.309	–3.743^*^	–2.619^*^	0.283
	LysM1	1.902^*^	3.682	1.132	1.600^*^	−4.186	–2.001^*^	0.209
	Int. LysM1-2	−0.139	0.006	−1.091	−0.934	0.264	0.500	0.658
	LysM2	1.334	−1.659	0.673	0.958	0.370	0.239	0.179
	Int. LysM2-3	–1.775^*^	−4.971	–3.959^***^	–3.836^***^	0.078	0.096	0.942^**^
	LysM3	−0.619	−1.815	–2.380^*^	–2.046^*^	0.019	−0.235	0.422
PsSym37	Overall	1.384	5.805	0.439	1.002	−6.786	−1.676	0.153
	Upstream LysM1	−0.030	−0.786	0.179	0.130	–2.342^*^	–2.161^*^	0.221
	LysM1	2.070^*^	3.403	0.474	1.196	−0.225	−0.169	0.237
	Int. LysM1-2	−0.268	−0.185	0.496	0.583	0.226	0.626	0.720
	LysM2	0.684	1.671	−0.557	−0.138	−0.769	−0.717	0.337
	Int. LysM2-3	1.613	4.028	0.956	1.374	−0.796	−0.966	0.550
	LysM3	1.398	3.516	0.474	0.860	−2.880	–2.905^*^	0.541
PsLykX	Overall	0.533	2.817	−1.478	−0.828	−9.689	–1.865^*^	0.169
	Upstream LysM1	0.146	0.234	−1.159	−0.752	0.909	0.650	0.270
	LysM1	0.107	1.213	–2.161^*^	−1.682	−2.954	−0.687	0.299
	Int. LysM1-2	0.683	1.263	0.496	0.643	0.239	0.662	0.691
	LysM2	1.241	0.595	0.474	0.741	–3.972^*^	–2.981^*^	0.297
	Int. LysM2-3	–	–	–	–	–	–	–
	LysM3	0.217	−0.437	−0.930	−0.632	–3.912^*^	–2.690^*^	0.229
MtLYK2	Overall	−1.064	–45.063^***^	0.119	−0.489	–20.660^**^	–4.385^**^	0.097^***^
	Upstream LysM1	−0.263	–10.208^***^	0.543	0.304	–2.984^*^	–2.452^*^	0.565^***^
	LysM1	−0.613	–5.803^*^	−1.354	−1.311	−0.491	−0.496	0.646^**^
	Int. LysM1-2	−0.811	−1.169	0.481	0.107	−1.862	–5.283^**^	0.949
	LysM2	−1.556	−2.436	−0.216	−0.929	–14.759^***^	–5.614^***^	0.674^***^
	Int. LysM2-3	−1.040	−0.762	0.815	0.264	0.211	0.317	0.839
	LysM3	−0.593	–8.523^**^	0.911	0.526	−0.774	−0.415	0.197
MtLYK3	Overall	−0.930	–155.381^***^	0.641	−0.032	–26.204^**^	4.255^**^	0.046^***^
	Upstream LysM1	−1.278	−4.046	−0.594	−1.092	0.405	0.563	0.685
	LysM1	−1.080	–22.651^***^	0.011	−0.436	–11.986^**^	–4.468^**^	0.113
	Int. LysM1-2	–	–	–	–	–	–	–
	LysM2	2.401^*^	−1.400	1.276	2.017^*^	−3.713	–3.157^*^	0.284
	Int. LysM2-3	−1.629	–7.577^***^	0.976	0.108	–5.802^***^	–6.069^***^	0.913^**^
	LysM3	–1.788^*^	–7.629^**^	0.430	0.430	–5.183^***^	−1.319	0.720^*^
MtLYK4	Overall	−0.100	–145.577^***^	0.656	0.522	–15.296^*^	–3.300^**^	0.040^***^
	Upstream LysM1	−0.576	−3.467	0.888	0.552	−1.050	−1.439	0.665^*^
	LysM1	−0.508	–11.353^**^	0.827	0.385	–7.239^**^	–3.520^**^	0.450^***^
	Int. LysM1-2	–	–	–	–	–	–	–
	LysM2	1.315	0.502	−0.090	0.630	−2.136	−1.391	0.302
	Int. LysM2-3	0.462	−0.253	0.637	0.901	0.098	0.325	0.449
	LysM3	−1.033	–6.292^*^	−0.230	−0.562	–5.186^**^	–3.271^*^	0.343

In case of statistically significant deviation from the neutral model, the values of criteria are given in bold and marked with asterisks accordingly to the P-value:

* P < 0.05;

** P < 0.01;

*** *P < 0.001*.

In *PsSym37*, the value of Tajima's D suggested a departure from the neutral model for the LysM1 domain, its positive value indicating balancing selection (in favor of several different variants, namely haplotypes A and B; Table 2). No deviations from neutral evolution were identified in other regions, except for a weak signal of positive selection upstream of LysM1 (FW-H criterion = −2.34, *P* = 0.046).

In *PsK1*, the Tajima's D value indicated balancing selection in LysM1, which appeared to be the most polymorphic part of this gene, and the region responsible for the segregation of two main haplotype groups A and B (Table 2). Interestingly, for the region between LysM2 and LysM3, Tajima's D value fell below the critical value, suggesting rapid spreading of a particular allelic state due to positive selection (selective sweep), or due to a recent population expansion. The FW-H value did not exceed the critical value, suggesting that population expansion has shaped the DNA sequence in this region. Thus, the low Tajima's D value should be attributed to genetic drift rather than to a selective advantage of a particular allelic state of *PsK1*. In other regions, no departures from the neutral evolution model were identified, except for a signal of positive selection upstream of LysM1 (FW-H criterion = −3.74, *P* = 0.018), similar to *PsSym37*.

*PsLykX*, like *M. truncatula MtLYK3* and *MtLYK4*, also showed a π_a_/π_s_ ratio >1 in the LysM1-encoding region (Table 4). Remarkably, a clear signal of positive selection was found in both the LysM2 and LysM3 domains (FW-H criterion below critical values; Figure 7), possibly indicating the importance of these parts for binding the Nod factor. Using a sliding window approach, declining values of modified Fu and Li's D and F criteria were detected at the LysM1 domain. However, in the absence of signals from powerful criteria (Fay and Wu's H), this was not considered as a footprint of positive selection. Instead, it could be attributed to a population expansion or a non-random selection of the samples in the sequence dataset.

In *Medicago*, all three examined genes had significantly low Fay and Wu's H values, indicating strong positive selection at the first exons (corresponding to the receptor parts of the LysM-RLKs). The strongest pressure was at LysM2 of MtLYK2 and at LysM1 of MtLYK3. In contrast to the pea sequences, the *M. truncatula* gene sequences showed significantly low values of Fu's Fs (Table 5), which is sensitive to population expansion. This implied that recent demographic processes have contributed to the molecular evolution of *M. truncatula* LysM-RLK genes. Interestingly, the similar pattern of nucleotide variant distribution in MtLYK3 and MtLYK4 genes led to comparable Tajima's D profiles in the sliding window analysis. The remarkable increase in Tajima's D at LysM2-encoding region (Figure 8) implied that there has been balancing selection at these sites. Meanwhile, the low value of Tajima's D at LysM3 of MtLYK3 was indicative of recent positive selection pressure at this domain. The FW-H and EW values also indicated that all three examined *M. truncatula* genes have undergone positive selection at the whole first exon, especially the LysM module-encoding parts. Recently, De Mita et al. (2014) pinpointed positive selection signatures at positions 43Q, 45R, and 77G in the MtLYK3 protein sequence. These results are consistent with the positive selection signatures found using other statistical models of nucleotide sequence diversity in the present study (sliding window midpoints 150 and 225; Figure 8).

## Discussion

Both natural and artificial selection tend to affect sequence variability at selected genomic loci and at neutral loci linked to them. These selection signatures are widely used to identify loci subjected to selection, thereby giving researchers an insight into the evolutionary process, as well as providing functional information about genes or genomic regions (Kreitman, 2000; Nielsen, 2005).

The most crucial step in the symbiosis between legume plants and nitrogen-fixing bacteria (rhizobia) is the initial mutual recognition of the macro- and micro-symbionts. The importance of this step should not be underestimated, as partnerships with ineffective bacteria can be lethal for plants growing in harsh environments. Thus, plants have developed multicomponent receptor systems for the bacterial signal to identify the most suitable and beneficial partner. As bacteria tend to evolve rapidly, the genes involved in their recognition should be “hot spots” under constant selective pressure. Therefore, one can expect to find footprints of selection in the nucleotide sequences of genes encoding legume symbiotic receptors. In this work, we studied the polymorphism of the first exon of three LysM-RLK genes in large set of pea genotypes, and compared them with their homologs in *M. truncatula*. Although all these genes appeared to be quite similar to each other, our data suggest that they differ significantly in their evolutionary patterns, consistent with the symbiotic partner recognition unique to each legume species.

A single amino acid replacement in the receptor part of the LysM-RLK can change its recognition of the Nod factor (as shown for *L. japonicus* LysM-RLK NFR5, Radutoiu et al., 2007). Therefore, we expected to find a relatively low level of polymorphism in the first exon (corresponding to the receptor domain) of the three LysM-RLK genes from the *Sym2* region. However, several “legal” haplotypes were detected for all three pea genes, implying that the structure of receptors may be variable. Such variability is possibly advantageous. Previously, Li et al. studied the polymorphism of the LysM-RLK gene *PsSym37* in 10 pea genotypes, and detected two groups of haplotypes (at the protein level) correlated with the ability of the plant to perceive Nod factors containing a C18:1 rather than a C18:4 (number of carbon atoms:number of unsaturated bonds) acyl group at the non-reducing end of molecule (Li et al., 2011). Despite our sample set being more than 10 times larger, we were unable to identify any new PsSym37 haplotypes in addition to those already discovered, which may indicate a certain fundamental base of this division among pea genotypes. However, it is hard to tell exactly how, where, and why these two symbiotic groups may have arisen, as the history of pea domestication, spreading, and crossing is long and unclear. Although our results indicate generally neutral evolution of *PsSym37*, the overall positive Tajima's D value indicates that the sequence dataset may be not uniform. Independent analyses of two lineages (haplotype groups A and B) may be required to identify footprints of positive selection.

Interestingly, *PsK1* shows a pattern of polymorphism similar to that of *PsSym37*, with two basic groups of haplotypes. However, the haplotypes do not correlate with any symbiotic feature of pea known so far. The function of *PsK1* is also unclear, although preliminary data (MSc. Anna Kirienko, Dr. Elena Dolgikh, personal communication) indicate that it may function prior to *PsSym37*, as suggested by phenotypic analyses of mutants. Since *PsK1* is similar to the *M. truncatula* gene *MtLYK2*, whose function is also unknown, it is possible that these proteins participate in the initial capture and recognition of the Nod factor, together with the “early” symbiotic LysM-RLKs PsSym10 (in pea) and MtNFP (in barrel medic). These kinases are characterized by an inactive kinase domain (lacking the activation loop; Arrighi et al., 2006), while this domain appears to be fully functional in PsK1 and MtLYK2. Therefore, the observed variability of the receptor part of PsK1 may be essential for recognition of diverse symbiotic bacteria.

The newly discovered pea LysM-RLK gene *PsLykX* is unique in that it has six equivalent major haplotype groups (each containing several genotypes) and about the same number of unique haplotypes (corresponding to a single genotype). This suggests that our sample of 90 *PsLykX* sequences does not cover all the possible “legal” allelic variants of this gene. Another important fact is the correlation of one of the identified *PsLykX* haplotypes with the “Afghan” phenotype. This makes *PsLykX* a promising candidate for *PsSym2*, and, among other things, may indicate that other *PsLykX* haplotypes are also related to nuances of perception of bacterial signals. The function of *MtLYK4*, the closest homologous *LYK* gene of *PsLykX*, is unclear, despite its significant similarity to *MtLYK3* both in its sequence and in its discernable selection signatures. In roots of *MtLYK4*-knockdown plants, infection thread morphology was affected but nodulation occurred normally (Limpens et al., 2003). Furthermore, evidence of positive selection in the LysM2 and LysM3 modules suggests that *MtLYK4* has recently acquired a new function, probably associated with the expansion of the *M. truncatula* population. Remarkably, the kinase domains of both putative PsLykX and MtLYK4 proteins lack the YAQ motif, which is inherent to symbiotic kinases in *L. japonicus* and is absent from non-symbiotic ones (Nakagawa et al., 2011). On the other hand, the receptor system in legumes with indeterminate nodules (e.g., pea and barrel medic) appears to be more complicated than that of legumes with determinate nodules, such as, *L. japonicus* (Ardourel et al., 1994). The presence of *PsLykX* transcripts in pea nodules, together with the *lyk4* RNA-interference phenotype in *M. truncatula* (disruption of infection process; Limpens et al., 2003), provide evidence for the participation of YAQ-lacking LysM-RLKs in indeterminate nodule development.

For all three genes, the haplotype distribution was not uniform among our samples, resulting in strictly correlating haplotype groups: for instance, group E of *PsLykX* (see Figure 6, Table 2) was found exclusively with group A2 of *PsK1* and B0 of *PsSym37*. However, considering that: (i) these genes are clustered within approximately 20 kb; (ii) the genotype sampling was not random; and (iii) the VIR collection does not represent a single population of pea, this non-uniformity can be explained by factors other than the evolutionary advantage of a particular haplotype combination, e.g., genetic linkage or founder effect. On the other hand, the fact that we observed so many haplotype combinations despite the close proximity of these genes suggests that there may be a selective advantage of symbiotic LysM-RLK haplotype shuffling in pea.

Despite the fact that the *LYK* region of *M. truncatula* and the *Sym2* region of pea are clearly syntenic (Gualtieri et al., 2002), it is nearly impossible to determine the orthologous relationships between genes contained in those regions. As shown in this work, LysM-RLK genes from these genomic regions are characterized by mutual mosaic similarity, with different parts of different genes being similar to each other both within a species and between species. Since the *LYK* and *Sym2* regions are represented primarily by gene clusters that apparently originated via multiplication of ancestral LysM-RLK gene(s), the genes in those regions have undergone concerted evolution (Liao, 1999) implying genetic conversion. This has led to the diversification of LysM-RLK gene clusters in different legume species. In other words, each cluster represents an “evolution crucible” unique to each species, where genes and gene parts have been shuffled, combined, or broken apart. Thus, divergent evolution may lead to the loss of earlier versions of genes in one species, and the retention of genes and acquisition of unique functions in another species through neofunctionalization. This is a promising explanation for the unique *PsSym2*^*A*^ phenotype that is not observed in any other legume species.

## Conclusion

As a complex multi-stage process, the symbiosis of legumes with rhizobia is not yet fully understood. There is still much to learn about the functions of many genes and the population genetics of the symbiosis. In *M. truncatula* (De Mita et al., 2011; Ho-Huu et al., 2012) and other legumes with well-studied genomes like *L. japonicus*, chickpea (*Cicer arietinum* L.), and soybean [*Glycine max* (L.) Merr.], surveys of large ecotype collections and association genetics analyses have been widely used to identify loci of interest (Kale et al., 2015; Li et al., 2017; Plekhanova et al., 2017). However, knowledge about the diversity of certain genes, especially those related to symbiosis, is still insufficient. The aim of this study was to help to fill this knowledge gap. Accordingly, we evaluated the genetic diversity of the pea LysM-RLK-encoding genes from the *Sym2* region and corresponding barrel medic genes from the *LYK* region, detected the signatures of positive, balancing and negative selection in the gene sequences, and compared the patterns of selection pressure affecting particular modules in the sequences.

The cultivated pea accessions analyzed in this work have been previously characterized to determine their productivity under symbiotic conditions (Borisov et al., 2002). Therefore, information on symbiotic LysM-RLK gene polymorphisms may be useful for studies on associations between gene variants and the formation of highly effective symbioses with beneficial microorganisms. Considering the importance and economic value of pea in modern agriculture, the value of such research activities should not be underestimated.

Analyses of the polymorphism of crucial symbiotic genes and the identification of selection signatures have allowed us to formulate new hypotheses about their roles in symbiosis, which will be tested experimentally in the near future. On the basis of our results, we propose that the newly discovered pea gene *PsLykX* could in fact be *PsSym2*, which is responsible for increased selectivity toward a symbiotic partner in plants with the characteristic phenotype of cv. Afghanistan. However, more detailed analyses are required to confirm that *PsLykX* is the elusive *PsSym2* gene. To confirm our hypothesis, the polymorphism of *PsLykX* should be studied in a larger sample of genotypes with high selectivity toward microsymbionts. In addition, it is important to analyze the phenotypes of lines with mutations in the *PsLykX* gene. The pea TILLING mutants collection (Dalmais et al., 2008) provides opportunities for such analyses. Complementation of the *PsLykX* mutations by either “European” or “Afghan” alleles resulting in nodulation or non-nodulation phenotypes would confirm the characteristics of particular groups of pea genotypes.

## Author contributions

VZ, IT, and LL designed and conceived the study; AS, AA, and AZ conducted experiments; AS, AZ, AA, VZ, and IT analyzed the data. AS, VZ, and AA drafted the manuscript, IT and LL critically revised the manuscript. All authors read and approved the final manuscript.

### Conflict of interest statement

The authors declare that the research was conducted in the absence of any commercial or financial relationships that could be construed as a potential conflict of interest.
