# Supplementary material for: Selection Signatures in the First Exon of Paralogous Receptor Kinase Genes from the Sym2 Region of the Pisum sativum L. Genome
Source: Front Plant Sci. 2017 Nov 14;8:1957. doi: 10.3389/fpls.2017.01957 (PMC5694491; doi:10.3389/fpls.2017.01957)
Supplement: Supplementary file 1 [file DataSheet1.DOCX]

Supplementary Material

Selection Signatures in the First Exon of Paralogous Receptor Kinase Genes from the *Sym2* Region of the *Pisum sativum* L. Genome

Sulima A.S., Zhukov V.A.^*^, Afonin A.A., Zhernakov A.I., Tikhonovich I.A., Lutova L.A.

*** Correspondence:** Vladimir Zhukov VZhukov@ARRIAM.ru

# Supplementary Figures

## Supplementary Figure 1. Visualization of similarity between (A) *PsSym37*, (B) *PsK1* and (C) *PsLykX* genes and the *LYK* region of *M. truncatula*.

The visualization was performed using ACT suite. The minimal identity cut-off was set to 70%.

# Supplementary Tables

## Supplementary Table 1. Primers used in the study.

| **BAC screening primers** | |
| --- | --- |
| K10_fw12 | 5' GCT TTC CTT CGG CAG CCA TTT TGT 3' |
| K10_RACE | 5' CAT TCA CAT GGG AAT GGA ATG TTG 3' |
| K1_fw3 | 5' GAA TGC TGT TCT GAA GAC AGG 3' |
| K1_rv10 | 5' GTC ATC ATC ACA ATC TTC AAA TG 3' |
| **Pea 1st exons primers** | |
| 99G_Sym37_F0 | 5' CAC TAG GAA CAT CCA TTC ATT 3' |
| 99G_Sym37_F1 | 5' ACA TGT CAA CTC TTA TTG TAG CC 3' |
| 99G_Sym37_F2 | 5' GAA TAG AAA AGC TAG GAA GTG TG 3' |
| 99G_Sym37_R0 | 5' CAA GTA CTT TTG ATC AGA ACC ACC 3' |
| 99G_Sym37_R1 | 5' ATT TAC CCA AGT CAC CGC GGG 3' |
| 99G_Sym37_R2 | 5' CTG AAG ATC AAA AGG TGG TGG 3' |
| 99G_K1_F1 | 5' GGA GTT GAA AGT GCA ATT AAG C 3' |
| 99G_K1_F2 | 5' CGT ATC CCA ACA TGC ATG TCT G 3' |
| 99G_K1_R1 | 5' GGA TAC AAA GGA ACA TAT TCT CC 3' |
| 99G_K1_R2 | 5' GCG AAG CCA TCA CAA TTC ATA CC 3' |
| 99G_LykX_F2 | 5' CCA CAT TCT CTA GTC AAA CAT 3' |
| LykX_Wh1_Fw1 | 5' CCC TCA GCA GCC ATT TTC AT 3' |
| LykX_Wh1_Fw2 | 5' CTC TCT TTC TTA TTG ACT AAA TG 3' |
| 99G_LykX_R1 | 5' TCT GTA AGT GAC AAC ATG CG 3' |
| 99G_LykX_R2 | 5' CGT CAG CGT AAG ATC CAA CC 3' |
| 99G_LykX_R3 | 5' GCA GAA GAT CGA AAG TTG ATG G 3' |
| **RACE *LykX* primers** | |
| LykX_5’.1 | 5' CAG AAG AAT TGA AGG CTG ACA CA 3' |
| LykX_5’.2 | 5' AGT AAA AAT GCA GGC ATG ACA TGG T 3' |
| LykX_5’.3 | 5' CAC ACA CTT TGA TTC CAC TTT GAA 3' |
| LykX_3’.1 | 5' GCA GAT GAC ACA GCT TGC GAA G 3' |
| LykX_3’.2 | 5' GAG ATC CGA AAG GAC GTC CAA GA 3' |
| LykX_3’.3 | 5' ATT GAG TCC CAT TGT GGA GCA TGA 3' |

## Supplementary Table 2. List of pea genotypes used in the study.

| **Name in VIR catalogue** | **Number** | **Known place of origin; cultivare/subspecies** | ***PsSym37* haplotype** | **Ps*K1* haplotype** | ***PsLykX* haplotype** |
| --- | --- | --- | --- | --- | --- |
| K-2429 | 1 | Algeria | A1 | A3 | A |
| K-2008 | 2 | Egypt (*Pisum jomardii*) | n/a | A1 | C |
| K-3429 | 3 | Egypt | B1 | A1 | C |
| K-7051 | 4 | Libya | A1 | A5 | A |
| K-8093 | 5 | Madagascar; "Felefone" | Unique | Unique | A |
| K-6468 | 6 | Sudan | A2 | B1 | A |
| K-7131 | 7 | Tunisia | n/a | n/a | n/a |
| K-7584 | 8 | Ethiopia | A0 | A1 | B |
| K-1836 | 9 | Africa | A0 | A5 | A |
| K-3856 | 10 | USA | B1 | A1 | C |
| K-3855 | 11 | Argentina | A0 | A3 | B |
| K-5012 | 12 | Argentina; "Ozo Negro" | n/a | B1 | D |
| K-8261 | 13 | Brazil; "Long drink" | A1 | B1 | A |
| K-8600 | 14 | - | A0 | A1 | D |
| K-8571 | 15 | Venezuela | B1 | A1 | C |
| K-693 | 16 | Canada | A0 | A5 | A |
| K-925 | 17 | USA | A0 | B1 | D |
| K-8572 | 18 | USA; "Mayfair" | n/a | A1 | C |
| K-7094 | 19 | Peru | A1 | Unique | A |
| K-6464 | 20 | Chile | A1 | B1 | A |
| K-1982 | 21 | Afghanistan | A0 | A3 | B |
| K-3266 | 23 | Armenia | A0 | A3 | B |
| K-2514 | 24 | Syria | A2 | B1 | B |
| K-2595 | 25 | Palestine | A1 | B1 | A |
| K-5493 | 26 | Iraq | A2 | B1 | A |
| K-2182 | 27 | Iran | n/a | n/a | n/a |
| K-958 | 28 | Turkmenistan | A1 | B1 | A |
| K-1251 | 29 | Tajikistan | A2 | B1 | B |
| K-7128 | 30 | Russia, Daghestan | A1 | A1 | A |
| K-188 | 31 | Pamir | A0 | B2 | C |
| K-1250 | 32 | Pamir | A2 | B1 | B |
| K-3540 | 33 | Japan | B0 | A2 | E |
| K-3034 | 34 | Japan | A2 | B1 | A |
| K-8599 | 35 | - | A1 | A2 | A |
| K-8456 | 36 | Burundi | A2 | B1 | A |
| K-1537 | 37 | Manchuria | n/a | A1 | C |
| K-4776 | 38 | Russia, Sakhalin | B0 | A2 | E |
| K-6373 | 39 | Mongolia | B0 | A4 | Unique |
| K-5494 | 40 | Mongolia | A0 | B2 | C |
| K-4788 | 41 | Mongolia | n/a | A2 | E |
| K-8702 | 42 | - | A0 | A3 | B |
| K-6063 | 43 | Kazakhstan | B0 | A2 | E |
| K-4662 | 44 | Russia, Primorsky Krai | B0 | A2 | E |
| K-3273 | 45 | Russia, Komi Republic | A0 | B1 | B |
| K-6875 | 46 | Russia, Krasnoyarsk Krai | B0 | A2 | E |
| K-7700 | 47 | Bangladesh | A2 | B1 | A |
| K-7034 | 48 | Nepal | A0 | A3 | B |
| K-8543 | 49 | England | A1 | A1 | C |
| K-1142 | 50 | France | B0 | A2 | E |
| K-1865 | 51 | India | A1 | B1 | A |
| K-1866 | 52 | India | A2 | B1 | A |
| K-6545 | 53 | India | n/a | n/a | n/a |
| K-1929 | 54 | India | A0 | A5 | A |
| K-2587 | 55 | Armenia | A0 | A5 | A |
| K-1985 | 56 | Georgia | A1 | B2 | A |
| K-3445 | 57 | Azerbaijan | n/a | A1 | C |
| K-8585 | 58 | Germany | A0 | A4' | D |
| К-5992 | 59 | Australia | B0 | A2 | E |
| K-8639 | 60 | - | B1 | A1 | C |
| K-1937 | 61 | Austria | B1 | A1 | C |
| K-8431 | 62 | Hungary | A1 | A5 | A |
| K-3980 | 63 | Georgia | B0 | A2 | Unique |
| K-4650 | 64 | Armenia | n/a | n/a | n/a |
| K-2593 | 65 | Cyprus | A1 | A2 | A |
| K-2175 | 66 | Turkey/Bulgaria | A1 | A4" | A |
| K-2176 | 67 | Czech Republic | A0 | A3 | B |
| K-8413 | 68 | Hungary | A2 | B1 | A |
| K-4148 | 69 | Finland | B1 | A1 | D |
| K-7826 | 70 | Poland | B1 | A1 | C |
| K-6139 | 71 | Albania | A0 | A1 | D |
| K-2174 | 72 | Bulgaria | A0 | A3 | B |
| K-2006 | 73 | Denmark (*P. arvense v. quadratum)* | n/a | B1 | D |
| K-3970 | 74 | Belgium | n/a | Unique | Unique |
| K-1022 | 75 | Germany (*Pisum jomardii*) | n/a | A1 | D |
| K-1027 | 76 | Germany | B1 | A1 | C |
| K-1025 | 77 | Germany | A1 | A1 | D |
| K-1143 | 78 | France | A0 | A3 | B |
| K-3140 | 79 | Spain | A1 | A1 | n/a |
| K-1818 | 80 | England/Italy | A1 | B1 | A |
| K-1930 | 81 | Italy | A0 | A5 | A |
| K-1693 | 82 | England | A0 | A4' | Unique |
| K-1699 | 83 | England | n/a | n/a | n/a |
| K-6883 | 84 | Uzbekistan | B0 | B1 | F |
| K-4170 | 85 | Latvia | n/a | Unique | Unique |
| K-116 | 86 | Latvia | A0 | A4' | n/a |
| K-1815 | 87 | Russia, Penza Oblast | B1 | A1 | B |
| K-3312 | 88 | Russia, Kirov Oblast | n/a | A4 | B |
| K-957 | 89 | Russia, Vologda Oblast | n/a | A2 | Unique |
| K-3063 | 90 | Russia, Leningrad Oblast | n/a | n/a | n/a |
| K-1685 | 91 | Sweden | B0 | A2 | E |
| K-3064 | 92 | Belarus | A2 | B1 | n/a |
| K-3324 | 93 | Ukraine, Luhansk Oblast | B0 | A4 | Unique |
| K-4789 | 94 | Ukraine, Drohobych Oblast | B1 | A1 | C |
| K-4108 | 95 | Ukraine, Lviv Oblast | A0 | A2 | n/a |
| K-3358 | 96 | Russia, Saratov Oblast | A0 | A1 | B |
| K-8246 | 97 | - | n/a | A3 | n/a |
| K-8522 | 98 | Russia, Moskow; "Troika" | A2 | B1 | A |
| K-8274 | 99 | France, "Vendevil" | A0 | A4' | B |
| K-8638 | 100 | Finland, "Panu" | A2 | B1 | A |
|  |  |  |  |  |  |
| Cameor |  | France | A1 | A4" | A |
| NGB2150 |  | Afghanistan | B0 | n/a | F |
| Finale |  | Canada | B1 | A1 | n/a |
| Sprint-2 |  | - | A0 | A4 | n/a |
| SGE |  | - | A0 | A4' | n/a |
| Sparkle |  | USA | A2 | B1 | n/a |
| Rondo |  | Netherlands | A2 | B1 | n/a |
| 2715 |  | - | n/a | B1 | n/a |
| 1238 |  | - | B0 | Unique | n/a |
| Frisson |  | France | A1 | n/a | n/a |

## Supplementary Table 3. Identity and similarity of *M. truncatula* MtLYK1-7, *P. sativum* PsSym37, PsK1 and PsLykX, *Lotus japonicus* LjNFR1 and *Arabidopsis thaliana* AtCERK1 putative proteins calculated for receptor part of the proteins by pairwise comparison.

| **Identity** | AtCERK1 | LjNFR1 | MtLYK1 | MtLYK2 | MtLYK3 | MtLYK4 | MtLYK5 | MtLYK6 | MtLYK7 | PsK1 | PsLykX | PsSym37 |
| --- | --- | --- | --- | --- | --- | --- | --- | --- | --- | --- | --- | --- |
| AtCERK1 | 100 | 47.2 | 43.5 | 45.8 | 44.9 | 44.4 | 46.5 | 45.1 | 48.9 | 44.2 | 45.6 | 45.9 |
| LjNFR1 | 47.2 | 100 | 49.3 | 69 | 64.5 | 61.7 | 53.5 | 45.5 | 51.4 | 65.1 | 66 | 66.7 |
| MtLYK1 | 43.5 | 49.3 | 100 | 47.7 | 45.6 | 44.2 | 66.5 | 58.9 | 64.5 | 46.7 | 49.8 | 48.6 |
| MtLYK2 | 45.8 | 69 | 47.7 | 100 | 72.3 | 70.4 | 47 | 44.9 | 48.1 | 74.2 | 74.6 | 77.5 |
| MtLYK3 | 44.9 | 64.5 | 45.6 | 72.3 | 100 | 95.3 | 47.7 | 43.9 | 48.8 | 75.5 | 72.8 | 75.9 |
| MtLYK4 | 44.4 | 61.7 | 44.2 | 70.4 | 95.3 | 100 | 46.3 | 43.9 | 47.9 | 74.1 | 70.9 | 74.1 |
| MtLYK5 | 46.5 | 52.3 | 66.5 | 47 | 47.7 | 46.3 | 100 | 55.5 | 64.2 | 49.3 | 51.2 | 49.3 |
| MtLYK6 | 45.1 | 45.5 | 58.9 | 44.9 | 43.9 | 43.9 | 55.5 | 100 | 57.3 | 44.1 | 46.7 | 44.5 |
| MtLYK7 | 48.9 | 51.4 | 64.5 | 48.1 | 48.8 | 47.9 | 64.2 | 57.3 | 100 | 50.5 | 52.3 | 52.4 |
| PsK1 | 44.2 | 65.1 | 46.7 | 74.2 | 75.5 | 74.1 | 49.3 | 44.1 | 50.5 | 100 | 77.9 | 78.2 |
| PsLykX | 45.6 | 66 | 49.8 | 74.6 | 72.8 | 70.9 | 51.2 | 46.7 | 52.3 | 77.9 | 100 | 77.5 |
| PsSym37 | 45.9 | 66.7 | 48.6 | 77.5 | 75.9 | 74.1 | 49.3 | 44.5 | 52.4 | 78.2 | 77.5 | 100 |
|  |  |  |  |  |  |  |  |  |  |  |  |  |
| **Similarity** | AtCERK1 | LjNFR1 | MtLYK1 | MtLYK2 | MtLYK3 | MtLYK4 | MtLYK5 | MtLYK6 | MtLYK7 | PsK1 | PsLykX | PsSym37 |
| AtCERK1 | 100 | 61.9 | 63 | 62.6 | 64.5 | 63.6 | 67.7 | 63.4 | 68.9 | 60 | 60.6 | 60.5 |
| LjNFR1 | 61.9 | 100 | 62 | 80.6 | 78.5 | 78.5 | 66.5 | 61.5 | 68.4 | 79.5 | 79.5 | 83.1 |
| MtLYK1 | 63 | 62 | 100 | 61.2 | 58.1 | 58.1 | 79.2 | 70.8 | 78.7 | 60.7 | 62.3 | 61.8 |
| MtLYK2 | 62.6 | 80.6 | 61.2 | 100 | 85 | 85 | 64.5 | 62.5 | 67.8 | 87.3 | 85.9 | 87.3 |
| MtLYK3 | 64.5 | 78.5 | 58.1 | 85 | 100 | 98.1 | 62.5 | 60.4 | 64.8 | 85.8 | 85 | 85.8 |
| MtLYK4 | 63.6 | 78.5 | 58.1 | 85 | 98.1 | 100 | 63 | 61.3 | 65.3 | 86.8 | 84.5 | 86.3 |
| MtLYK5 | 67.7 | 66.2 | 79.2 | 64.5 | 62.5 | 63 | 100 | 72.5 | 79.2 | 64.8 | 63.3 | 63.4 |
| MtLYK6 | 63.4 | 61.5 | 70.8 | 62.5 | 60.4 | 61.3 | 72.5 | 100 | 72.5 | 62.1 | 63.2 | 61.1 |
| MtLYK7 | 68.9 | 68.4 | 78.7 | 67.8 | 64.8 | 65.3 | 79.2 | 72.5 | 100 | 68.9 | 69.6 | 67.9 |
| PsK1 | 60 | 79.5 | 60.7 | 87.3 | 85.8 | 86.8 | 64.8 | 62.1 | 68.9 | 100 | 87.8 | 89.1 |
| PsLykX | 60.6 | 79.5 | 62.3 | 85.9 | 85 | 84.5 | 63.3 | 63.2 | 69.6 | 87.8 | 100 | 88.7 |
| PsSym37 | 60.5 | 83.1 | 61.8 | 87.3 | 85.8 | 86.3 | 63.4 | 61.1 | 67.9 | 89.1 | 88.7 | 100 |

The “heat map” coloring indicates the degree of identity and similarity.

## Supplementary Table 4. Identity and similarity of *M. truncatula* MtLYK1-7, *P. sativum* PsSym37, PsK1 and PsLykX, *Lotus japonicus* LjNFR1 and *Arabidopsis thaliana* AtCERK1 putative proteins calculated for kinase part of the proteins by pairwise comparison.

| **Identity** | AtCERK1 | LjNFR1 | MtLYK2 | MtLYK3 | MtLYK4 | MtLYK5 | MtLYK6 | MtLYK7 | PsK1 | PsLykX | PsSym37 |
| --- | --- | --- | --- | --- | --- | --- | --- | --- | --- | --- | --- |
| AtCERK1 | 100 | 30.80 | 30.30 | 31.10 | 37.60 | 37.50 | 30.00 | 30.70 | 31 | 34.50 | 30.80 |
| LjNFR1 | 30.80 | 100 | 83.00 | 84.70 | 56.30 | 55.70 | 58.80 | 60.50 | 86 | 57.90 | 84.20 |
| MtLYK2 | 30.30 | 83 | 100 | 85.50 | 54.60 | 54.30 | 59.20 | 60.30 | 84.40 | 59.40 | 83.10 |
| MtLYK3 | 31.10 | 84.70 | 85.50 | 100 | 51.70 | 51.80 | 59.20 | 61.40 | 89 | 59 | 87.70 |
| MtLYK4 | 37.60 | 56.30 | 54.60 | 51.70 | 100 | 84.80 | 53.90 | 55 | 54.90 | 69.60 | 54.60 |
| MtLYK5 | 37.50 | 55.70 | 54.30 | 51.80 | 84.80 | 100 | 55.30 | 55 | 55.30 | 71.30 | 55.10 |
| MtLYK6 | 30 | 58.80 | 59.20 | 59.20 | 53.90 | 55.30 | 100 | 67.10 | 59.40 | 53.80 | 57.90 |
| MtLYK7 | 30.70 | 60.50 | 60.30 | 61.40 | 55 | 55 | 67.10 | 100 | 61.20 | 52.90 | 60.50 |
| PsK1 | 31 | 86 | 84.40 | 89 | 54.90 | 55.30 | 59.40 | 61.20 | 100 | 58.80 | 90.50 |
| PsLykX | 34.50 | 57.90 | 59.40 | 59 | 69.60 | 71.30 | 53.80 | 52.90 | 58.80 | 100 | 58.80 |
| PsSym37 | 30.80 | 84.20 | 83.10 | 87.70 | 54.60 | 55.10 | 57.90 | 60.50 | 90.50 | 58.80 | 100 |
|  |  |  |  |  |  |  |  |  |  |  |  |
| **Similarity** | AtCERK1 | LjNFR1 | MtLYK2 | MtLYK3 | MtLYK4 | MtLYK5 | MtLYK6 | MtLYK7 | PsK1 | PsLykX | PsSym37 |
| AtCERK1 | 100 | 39.6 | 37.5 | 38 | 40.9 | 41.5 | 37 | 37.7 | 38.2 | 38.9 | 37.3 |
| LjNFR1 | 39.6 | 100 | 90 | 90.3 | 67.4 | 67 | 72 | 73.7 | 91 | 68.7 | 88.3 |
| MtLYK2 | 37.5 | 90 | 100 | 91.9 | 67.8 | 68.4 | 73.5 | 73.7 | 91.7 | 71.5 | 89.5 |
| MtLYK3 | 38 | 90.3 | 91.9 | 100 | 64.6 | 65.5 | 72 | 74.5 | 94.1 | 70.6 | 91.9 |
| MtLYK4 | 40.9 | 67.4 | 67.8 | 64.6 | 100 | 90.4 | 67.8 | 66.2 | 66.3 | 77.6 | 68 |
| MtLYK5 | 41.5 | 67 | 68.4 | 65.5 | 90.4 | 100 | 69.2 | 67.3 | 67.8 | 80 | 68.9 |
| MtLYK6 | 37 | 72 | 73.5 | 72 | 67.8 | 69.2 | 100 | 77.2 | 72.7 | 67.2 | 68.9 |
| MtLYK7 | 37.7 | 73.7 | 73.7 | 74.5 | 66.2 | 67.3 | 77.2 | 100 | 73.9 | 66.1 | 73 |
| PsK1 | 38.2 | 91 | 91.7 | 94.1 | 66.3 | 67.8 | 72.7 | 73.9 | 100 | 70.8 | 94.1 |
| PsLykX | 38.9 | 68.7 | 71.5 | 70.6 | 77.6 | 80 | 67.2 | 66.1 | 70.8 | 100 | 70.1 |
| PsSym37 | 37.3 | 88.3 | 89.5 | 91.9 | 68 | 68.9 | 68.9 | 73 | 94.1 | 70.1 | 100 |

The “heat map” coloring indicates the degree of identity and similarity.
