# Supplementary figures and images for: Selection Signatures in the First Exon of Paralogous Receptor Kinase Genes from the Sym2 Region of the Pisum sativum L. Genome
Source: Front Plant Sci. 2017 Nov 14;8:1957. doi: 10.3389/fpls.2017.01957 (PMC5694491; doi:10.3389/fpls.2017.01957)

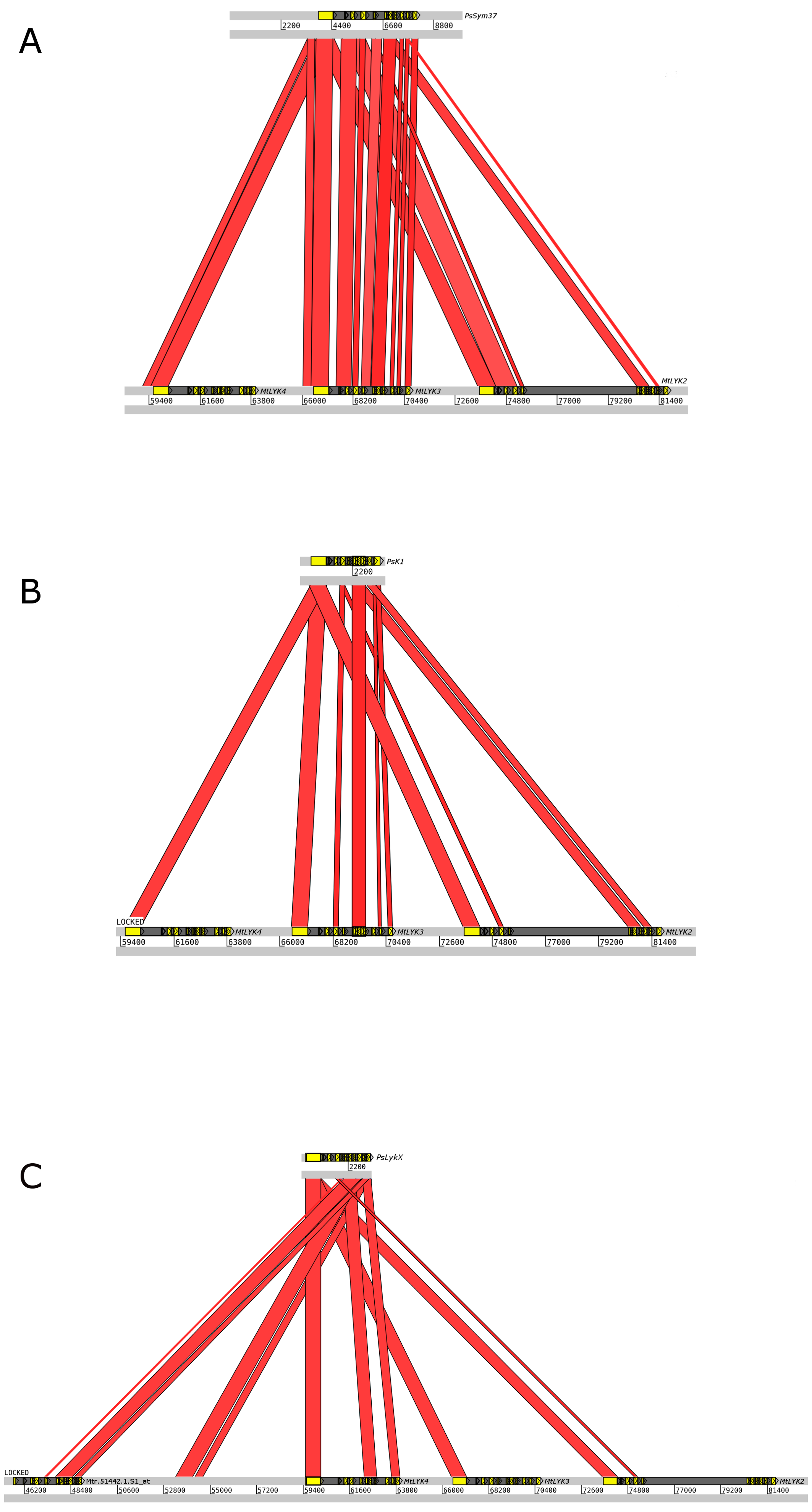

Supplement: Supplementary file 2 [file Image1.TIFF]
